# New Diketopiperazines from a Marine-Derived Fungus Strain *Aspergillus versicolor* MF180151

**DOI:** 10.3390/md17050262

**Published:** 2019-05-02

**Authors:** Jiansen Hu, Zheng Li, Jieyu Gao, Hongtao He, Huanqin Dai, Xuekui Xia, Cuihua Liu, Lixin Zhang, Fuhang Song

**Affiliations:** 1 Chinese Academy of Sciences Key Laboratory of Pathogenic Microbiology and Immunology, Institute of Microbiology, Chinese Academy of Sciences, Beijing 100101, China; huzxcv10@126.com (J.H.); bluewave2015@sina.com (Z.L.); jieyu_gao@163.com (J.G.); hehongtao2010@live.cn (H.H.); huanqindai@gmail.com (H.D.); 2 University of Chinese Academy of Sciences, Beijing 100049, China; 3 School of Food and Biological Engineering, Hefei University of Technology, Hefei 230009, China; 4 Key Biosensor Laboratory of Shandong Provinde, Biology Institute, Qilu University of Technology (Shandong Academy of Sciences), Jinan 250013, China; xiaxk@sdas.org; 5 State Key Laboratory of Bioreactor Engineering, East China University of Science of Technology, Shanghai 200237, China

**Keywords:** marine-derived fungus, *Aspergillus versicolor*, diketopiperazine, antibacterial

## Abstract

Six new diketopiperazines, (±)-7,8-epoxy-brevianamide Q ((±)-**1**), (±)-8-hydroxy-brevianamide R ((±)-**2**), and (±)-8-epihydroxy-brevianamide R ((±)-**3**), together with four known compounds, (±)-brevianamide R ((±)-**4**), versicolorin B (**5**) and averufin (**6**), were isolated from a marine-derived fungus strain *Aspergillus versicolor* MF180151, which was recovered from a sediment sample collected from the Bohai Sea, China. The chemical structures were established by 1D- and 2D-NMR spectra and HR-ESI-MS. **1** is the first sample of brevianamides with an epoxy moiety. Their bioactivities were evaluated against *Candida albican*s, *Bacillus subtilis*, *Staphylococcus aureus*, methicillin-resistant *S. aureus*, *Pseudomonas aeruginosa*, and Bacillus Calmette-Guérin. Compounds **1**–**4** showed no activities against the pathogens, and compounds **5** and **6** showed moderate activities against *S. aureus* and methicillin-resistant *S. aureus*.

## 1. Introduction

Marine-derived fungi are revealed to be excellent resources for novel secondary metabolites and many lead compounds have been characterized for drug development [1,2,3]. *Aspergillus versicolor*, a slow-growing filamentous fungus, normally are found in air, soil, marine sediment, corrupted plants, and agricultural products. Previous chemical investigations on the fungus *Aspergillus versicolor* from different environments have resulted in the identification of new secondary metabolites with a variety of structures, such as alkaloids [4,5,6,7,8,9], anthraquinones [10,11,12,13,14], xanthones [15,16,17,18,19], diphenyl ethers [20,21], lactones [22,23,24,25,26], peptides [27], polyketide [28], terpenoids [29,30], and varicuothiols [31].

**Figure 1:**
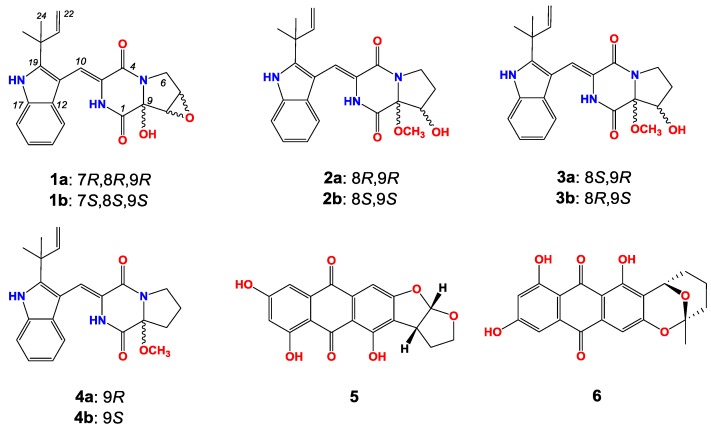
The structures of compounds **1**–**6**.

During our continuous screening of new secondary metabolites from marine *Aspergillus versicolor*, six new diketopiperazines ((±)-**1**–(±)-**3**) named as (±)-7,8-epoxy-brevianamide Q, (±)-8-hydroxy-brevianamide R, and (±)-8-epihydroxy-brevianamide R along with four known compounds (±)-brevianamide R, versicolorin B and averufin ((±)-**4**–**6**, Figure 1) were isolated from the culture material of a marine-derived fungus strain *Aspergillus versicolor* MF180151. Compound **1** is the first sample of brevianamides with an epoxy moiety. In this paper, we describe the fermentation, isolation, structure elucidation and preliminary bioactivities of these compounds.

## 2. Results

### 2.1. Characterization and Identification of the Isolated Strain MF180151

The strain MF180151 was isolated from a marine sediment sample from the Bohai Sea, China. The identification of the strain was performed based on the morphology and phylogenetic analysis.

**Figure 2:**
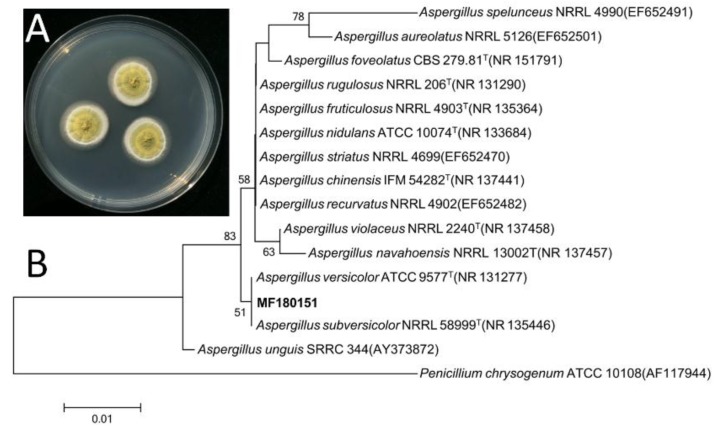
Morphology and neighbor-joining phylogenetic tree of strain MF180151. A: The morphology of the strain MF180151; B: The neighbor-joining phylogenetic tree of strain MF180151, numbers at nodes indicate levels of bootstrap support (%) based on a neighbor-joining analysis of 1,000 resampled datasets; only values >50% are given.

The ITS gene region of ribosomal DNA of the strain was PCR-amplified and sequenced. By comparing the ITS sequence to GenBank, it was indicated that the strain MF180151 belonged to the genus *Aspergillus* and shared a highest similarity with *Aspergillus versicolor* (99.66%). The phylogenetic tree based on ITS gene sequence revealed that the strain MF180151 formed a distinct phylogenetic cluster with *A. versicolor* (Figure 2) with a bootstrap value above 95%.

### 2.2. Structure Elucidation

**Figure 3:**
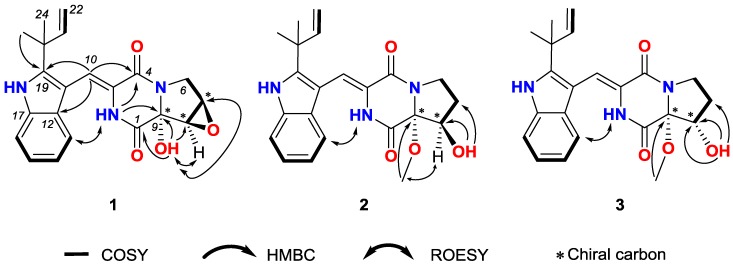
COSY, Key HMBC and ROESY correlations of compounds **1**–**3**.

(±)-7,8-Epoxy-brevianamide Q ((±)-**1**) were isolated as a light yellow amorphous powder. The molecular formula of **1** was established as C_21_H_21_N_3_O_4_ by HRESIMS (*m/z* 380.1608 [M + H]^+^ showed in Figure S7, calcd for C_21_H_22_N_3_O_4_: 380.1605). The planar structure of **1** was determined by 1D and 2D NMR spectra analyses, including ^1^H, ^13^C, ^1^H-^1^H homonuclear correlated spectroscopy (COSY), heteronculear single quantum coherence (HSQC) and heteronuclear multiple bond correlation (HMBC, Figures S1–S5). The ^1^H and ^13^C NMR data of **1** is tabulated in Table 1, which revealed the moieties of indole, diketopiperazine, prenyl, and one isolated double bond. Further analyses of 2D NMR data confirmed these moieties. Additionally, the HMBC correlations from the methyl groups (*δ*_H_ 1.50 and 1.45, H_3_-23 and H_3_-24) to C-19 (*δ*_C_ 144.6), C-20 (*δ*_C_ 39.0) and C-21 (*δ*_C_ 145.1) suggested that C-20 of the prenyl was attached to C-19 of the indole moiety. The HMBC crossing peaks from H-10 (*δ*_H_ 7.02) to C-12 (*δ*_C_ 126.2), C-19 (*δ*_C_ 144.6) and C-4 (*δ*_C_ 160.2) revealed that the diketopiperazine and indole moieties were connected by double bond of C-3 (*δ*_C_ 124.5) and C-10 (*δ*_C_ 113.4). The connectivity among C-1, C-9 and C-8 was confirmed by the HMBC correlations from 9-OH (*δ*_H_ 7.54) to C-1 (*δ*_C_ 163.1), C-9 (*δ*_C_ 86.0) and C-8 (*δ*_C_ 57.4). Thus the planar structure of **1** was assigned as shown in Figure 1. The rotating frame overhauser effect spectroscopy (ROESY, Figure S6) correlation between 2-NH (*δ*_H_ 9.37) and H-13 (*δ*_H_ 7.32) suggested the *cis* form of the double bond between C-3 and C-10. The ROESY signal from 9-OH (*δ*_H_ 7.54) to H-7 (*δ*_H_ 3.95)/H-8 (*δ*_H_ 3.93) revealed the relative configurations of **1** (Figure 3).

(±)-8-Hydroxy-brevianamide R ((±)-**2**) were isolated as a light yellow amorphous powder. The molecular formula of **2** was established as C_22_H_25_N_3_O_4_ by HRESIMS (*m/z* 396.1912 [M + H]^+^ showed in Figure S15, calcd for C_22_H_26_N_3_O_4_: 396.1918). Analyses of the ^1^H, ^13^C, COSY and HSQC NMR data (Table 1, Figures S9–S11, S13) revealed that **2** possessed the same carbon skeleton as that of **1**. By comparing the NMR data of **2** with those of brevianamide U [9] and brevianamide R [32], it was revealed that **2** was methylated at the 9-hydroxyl group of brevianamide U, which was confirmed by the HMBC (Figure S12) crossing peak from 9-OMe (*δ*_H_ 3.23) to C-9 (*δ*_C_ 94.5). The 8-hydroxyl group was confirmed by the HMBC correlations from 8-OH (*δ*_H_ 5.52) to C-7 (*δ*_C_ 28.4), C-8 (*δ*_C_ 74.0) and C-9 (*δ*_C_ 94.5). Thus, the planer structure of **2** was assigned. In the ROESY (Figure S14) spectrum of **2**, the correlation between 2-NH (*δ*_H_ 9.25) and H-13 (*δ*_H_ 7.14) suggested the *cis* form of double bond between C-3 and C-10. And the ROESY correlations between 9-OMe (*δ*_H_ 3.23) and H-8 (*δ*_H_ 4.22), and the absence between 9-OMe (*δ*_H_ 3.23) and 8-OH (*δ*_H_ 5.52) indicated that 9-OMe and H-8 were *cis* form. Therefore, the relative configurations of **2** was established as shown in Figure 3.

(±)-8-Epihydroxy-brevianamide R ((±)-**3**) were isolated as a light yellow amorphous powder. The molecular formula of **3** was established as C_22_H_25_N_3_O_4_ by HRESIMS (*m/z* 396.1921 [M+H]^+^ showed in Figure S22, calcd for C_22_H_26_N_3_O_4_: 396.1918). By comparing the ^1^H, ^13^C NMR, COSY and HSQC data (Table 1, Figures S17–S19, S21) of **3** with those of **2**, it is revealed that **3** possessed the similar structure as that of **2**. Analyses of the 2D NMR data suggested the same planer structure of **3** and **2**. The 8-hydroxyl group was confirmed by the HMBC (Figure S20) correlations from 8-OH (*δ*_H_ 5.14) to C-7 (*δ*_C_ 27.6), C-8 (*δ*_C_ 73.6) and C-9 (*δ*_C_ 87.1). And the HMBC signal from 9-OMe (*δ*_H_ 3.42) to C-9 (*δ*_C_ 87.1) revealed the methyoxyl group at C-9. In the ROESY (Figure S22) spectrum of **3**, the correlation between 2-NH (*δ*_H_ 9.35) and H-13 (*δ*_H_ 7.22) suggested the *cis* form of double bond between C-3 and C-10. By comparison the chemical shift of C-9 for **3** with that of **2**, **3** was established as an epimer of **2**, with the relative configuration of 9-OCH_3_ and 8-OH being *cis* and named as (±)-8-epihydroxy-brevianamide R which was shown in Figure 3.

(±)-**1**–(±)-**3** did not show significant CD spectra absorption (Figure S8, S16, and S23) and optical rotations, ${\left[\alpha \right]}_{D}^{25}$ +3.00 (*c* 0.1, CH_3_OH) for (±)-**1**, ${\left[\alpha \right]}_{D}^{25}$ +1.00 (*c* 0.1, CH_3_OH) for (±)-**2**, and ${\left[\alpha \right]}_{D}^{25}$ +2.00 (*c* 0.1, CH_3_OH) for (±)-**3**, which indicated that (±)-**1**–(±)-**3** were racemic mixtures.

Additionally, the structures of the three known compounds were identified as (±)-brevianamide R ((±)-**4**) [32], versicolorin B (**5**) [33] and averufin (**6**) [34] based on its HRESIMS, ^1^H NMR, and ^13^C NMR data and comparing with previous reports.

### 2.3. Biological Activities

The biological activity of those compounds were evaluated against pathogens of Bacillus Calmette-Guérin (BCG), *C. albicans*, *B. subtilis*, *S. aureus*, methicillin-resistant *S. aureus* (MRSA), and *P. aeruginosa*. The new diketopiperazines (±)-**1**–(±)-**3** and (±)-brevianamide R ((±)-**4**) showed no significant antibacterial activities against those pathogens. Versicolorin B (**5**) exhibited moderate activities against *S. aureus* and MRSA with the MIC values of 6.25 µg/mL and 12.5 µg/mL. Simultaneously, averufin (**6**) exhibited moderate activities against *S. aureus* and MRSA with the MIC values of 6.25 µg/mL and 25 µg/mL (Table 2).

## 3. Discussion

Brevianamides belong to a class of naturally occurring 2,5-diketopiperazine alkaloids, which are mainly produced by fungi of *Penicillium* and *Aspergillus* [6,9,32,35,36]. In this research, three pairs of new brevianamides ((±)-**1**–(±)-**3**) were isolated and their relative configurations were elucidated according to the 1D, 2D NMR, HRESIMS, UV. But, the specific optical rotation analysis and CD showed that these compounds were racemic mixtures. In more than 24 brevianamides, the hydroxy-substitution were mainly occurred at C-8 or/and C-9. In our research, (±)-7,8-epoxy-brevianamide Q ((±)-**1**) was discovered as the first brevianamide analogues with an epoxy substitution. Compounds (±)-**1**–(±)-**4** did not exhibit antifungal and antibacterial activities against *C. albicans*, *B. subtilis*, *S. aureus*, MRSA, *P. aeruginosa* and Bacillus Calmette-Guérin (MIC >100 µg/mL). Versicolorin B (**5**) exhibited moderate activities against *S. aureus* and MRSA with the MIC values of 6.25 µg/mL and 12.5 µg/mL. Simultaneously, averufin (**6**) exhibited moderate activities against *S. aureus* and MRSA with the MIC values of 6.25 µg/mL and 25 µg/mL.

## 4. Materials and Methods

### 4.1. General Experimental Details

Specific optical rotations ([α]D) were measured on AntonPaar MCP 200 polarimeter (Anton Paar GmbH, Graz, Austria) in a 100 × 2 mm cell. CD spectra were measured on Chirascan spectropolarimeter (Applied Photophysics Ltd., Leatherhead, UK) in 1 mm quartz cells. UV-visible spectra were obtained on a Cary 50 spectrophotometer (Varian Inc., Palo Alto, CA, USA) in 1 cm quartz cells. NMR spectra were obtained on a Bruker Avance DRX600 spectrometer (Bruker BioSpin Corp., Billerica, MA, USA) at 600 MHz for ^1^H and ^13^C NMR. Chemical shifts were calibrated using residual solvent signals (DMSO-*d*_6_: *δ*_C_ 39.5, *δ*_H_ 2.50). High-resolution electrospray ionization mass spectrometry measurements were obtained on an Agilent 6520QTOF mass spectrometer (Aglient Technologies Inc., Santa Clara, CA, USA). TLC H silica (Qingdao Marine Chemical Factory, Qingdao, China), Sephadex LH-20 (GE Healthcare BioSciences AB, Uppsala, Sweden) were used for purification. Analytical and semipreparative HPLC was performed using Agilent 1100 or 1200 Series separations modules equipped with Agilent 1100 or 1200 Series diode array detectors and fraction collectors, controlled using ChemStation Rev.B.02.01 (Aglient Technologies Inc., Santa Clara, CA, USA).

### 4.2. Fungal Culture and Identification

The strain MF180151 was isolated from a marine sediment sample from the Bohai Sea, China. It was incubated on potato dextrose agar (PDA) plate consisting (0.4% potato starch, 2% dextrose, and 2% agar) at 28 °C. The identification was performed based on the morphology and phylogenetic analysis. The whole genomic DNA of the strain was extracted using the E.Z.N.A. kit (Omega Bio-Tek, Norcross, GA, USA). A pair of primers (ITS4: 5″-TCCTCCGCTTATTGATATGC-3″; ITS5: 5″-GGAAGTAAAAGTCGTAACAAGG-3″) was used to amplify the ITS region of MF180151. PCR amplification (50.0 μL final volume: 25 μL 2 × Taq Master Mix, 2 μL of 10 μM of each primer, 5.0 μL DNA template and 16 μL ddH_2_O) of the ITS sequence was performed on Bio-gener PCR Thermal Cycler with the initial denaturation at 95 °C for 3 min, 32 cycles of denaturation (94 °C, 15 s), annealing (60 °C, 15 s), and elongation (72 °C, 60 s), and a final elongation at 72 °C for 5 min. After multiple alignments of ITS sequence of the related species by CLUSTAL W [37], phylogenetic analysis was constructed using neighbor-joining method with bootstrap values based on 1000 replications by MEGA 5.0 [38,39].

The strain was deposited at the Institute of Microbiology, Chinese Academy of Sciences. The nucleotide sequences of ITS gene (accession number MK680178) of *A. versicolor* MF180151 were deposited in GenBank.

### 4.3. Fermentation, Extraction and Isolation

The strain MF180151 was cultured on potato dextrose agar plate at 28 °C for 7 days. Mature colonies were cut into small pieces (about 1 cm^2^) under aseptic conditions. Then, three piece of the strain was inoculated into three 250 mL conical flasks, each containing 40 mL of liquid medium consisting of potato infusion (20%), glucose (2.0%), artificial sea salt (3.5%) and distilled artificial seawater, at 28 °C for 3 d on a rotary shaker at 160 rpm. An aliquot (5 mL) of the resultant seed culture was inoculated into twelve 1 L conical flasks, each containing solid medium consisting of rice (100 g) and artificial seawater (30 mL), and the flasks were incubated stationary for 28 d at 20 °C.

The whole culture media was extracted exhaustively with EtOAc:MeOH (80:20). The combined extracts were reduced to dryness *in vacuo* and the residue was partitioned between EtOAc and H_2_O. The EtOAc layer (10.3 g) was subjected to a normal phase silica gel chromatography (60 × 80 mm column, TLC H silica) using a stepwise gradient of 50–100% hexane/CH_2_Cl_2_ and then 0–100% MeOH/CH_2_Cl_2_ to afford 15 fractions (500 mL each). The ninth fraction was chromatographed over a Sephadex LH-20 column (700 × 30 mm) using an isocratic elution of hexane: CH_2_Cl_2_:MeOH (5:5:1), to give four subfractions (F1–F4; 100 mL each). Subfraction F3 (205.6 mg) was further purified by HPLC (Agilent Zorbax SB-C18 250 × 9.4 mm, 5 μm column, 3.0 mL/min, isocratic 65% MeCN/H_2_O) to yield **1** (3.5 mg), **2** (4.2 mg), **3** (5.7 mg), and **4** (4.8 mg). The eighth fraction was purified by HPLC (Agilent Zorbax SB-C18 250 × 9.4 mm, 5 μm column, 3.0 mL/min, isocratic 60% MeCN/H_2_O) to yield **5** (3.9 mg) and **6** (6.7 mg).

### 4.4. Biological Activities

The biological activities of isolated compounds were assessed according to the previous report [9]. A panel of human pathogens were used for the assay, including *B. subtilis* (ATCC 6633), *S. aureus* (ATCC 6538), methicillin-resistant *S. aureus* (clinical strain from Chaoyang Hospital, Beijing, China), and Bacillus Calmette–Guérin (Pasteur 1173P2), *P. aeruginosa* (ATCC 15692), and fungus *C. albican*s (SC 5314).

For general antimicrobial assays, a single colony which was incubated on an LB agar overnight at 37 °C was picked up and suspended in Mueller-Hinton Broth to approximately 1 × 10^4^ cfu/mL. For anti-*C. albican*s assay, a colony of *C. albicans* incubated on a YPD agar plate was picked and suspended in RPMI 1640 to a concentration of 1 × 10^4^ cfu/mL. A twofold serial dilution of each compound to be tested was prepared, and an aliquot of each dilution (2μL) was added to a 96-well flat-bottom microtiter plate (Greiner). Vancomycin, ciprofloxacin and ketoconazole were used as the positive control and DMSO as the negative control. An aliquot (78 µL) of suspension was then added to each well (to give final compound concentrations of 100 to 0.78 µg/mL in 2.5% DMSO) and the plate was incubated at 37 °C aerobically for 16 h. The MIC was defined as the minimum concentration of the compound that prevented visible growth of the tested bacteria. All the experiments were performed in triplicate.

The strain Bacillus Calmette–Guérin (Pasteur 1173P2) used for the anti-BCG assay was transformed with green fluorescent protein (GFP) constitutive expression plasmid pUV3583c with direct readout of fluorescence as a measure of bacterial growth. The strain was incubated to mid log phase (7 d) at 37 °C in Middle brook 7H9 broth (40 mL; Difco) supplemented with 10% OADC enrichment (Becton Dickinson), 0.05% Tween-80 and 0.2% glycerol and then diluted to an OD_600_ of 0.025 with broth. Aliquots (80 μL) of the bacterial suspension were added to each well of the 96-well micro plates (clear flat-bottom), followed by adding compounds (2 μL in DMSO), which were serially twofold diluted. Isoniazid served as positive control and DMSO as negative control. The plate was incubated at 37 °C for 3 days, and GFP fluorescence was measured with Multi-label Plate Reader using the bottom read mode, with excitation at 485 nm and emission at 535 nm. MIC is defined as the minimum concentration of drug that inhibits more than 90% of bacterial growth reflected by fluorescence value.

## Figures and Tables

**Table 1:** ^1^H and ^13^C NMR data (600 MHz, DMSO-*d*_6_) for compounds **1**–**3**.

Position	1		2		3	
	*δ*_C_	*δ*_H_, mult (*J* in Hz)	*δ*_C_	*δ*_H_, mult (*J* in Hz)	*δ*_C_	*δ*_H_, mult (*J* in Hz)
1	163.1		160.9		162.9	
2		9.37, s		9.25, s		9.35, s
3	124.5		124.7		124.7	
4	160.2		159.3		160.1	
6a	45.6	3.52, d (13.2)	43.4	3.42, ddd (12.0, 10.2, 1.8)	40.6	3.47, m
6b		3.94, overlap		3.91, ddd (12.0, 8.4, 8.4)		
7a	51.1	3.95, overlap	28.4	2.13, m	27.6	2.12, m
7b				1.76, ddd (13.2, 8.4, 1.8)		1.86, dq (12.0, 9.6)
8	57.4	3.93, overlap	74.0	4.22 dd (4.8, 4.8)	73.6	4.29, ddd (14.4, 6.0, 3.0)
8-OH				5.52, d (4.8)		5.14, d (6.0)
9	86.0		94.5		87.1	
9-OH		7.54, s				
9-OMe			50.6	3.23, s	51.8	3.42, s
10	113.4	7.02, s	112.0	7.04, s	112.6	7.03, s
11	104.0		103.3		103.7	
12	126.2		126.2		126.2	
13	119.7	7.32, d (7.8)	118.6	7.14, d (7.8)	119.0	7.22, d (7.8)
14	119.3	7.00, dd (7.8, 7.8)	119.5	7.02, dd (7.8, 7.8)	119.4	7.02, dd (7.8, 7.8)
15	120.7	7.08, dd (7.8, 7.8)	120.8	7.10, dd (7.8, 7.8)	120.8	7.09, dd (7.8, 7.8)
16	111.4	7.41, d (7.8)	111.7	7.43, d (7.8)	111.6	7.42, d (7.8)
17	135.1		135.2		135.1	
18-NH		11.06, s		11.09, s		11.09, s
19	144.6		144.3		144.5	
20	39.0		39.0		39.0	
21	145.1	6.08, dd (17.4, 10.8)	145.1	6.08, dd (17.4, 10.8)	145.1	6.07, dd (17.4, 10.8)
22a	111.7	5.05, d (17.4)	111.7	5.04, d (17.4)	111.7	5.04, d (17.4)
22b		5.06, d (10.8)		5.06, d (10.8)		5.06, d (10.8)
23	27.4	1.50, s	27.4	1.49, s	27.4	1.49, s
24	27.8	1.45, s	27.7	1.47, s	27.7	1.45, s

**Table 2:** Antimicrobial Activities of **1**–**6**.

Organism (strain)	Minimum Inhibitory Concentration (μg/mL)						
	1	2	3	4	5	6	Control
Bacillus Calmette–Guérin (Pasteur 1173P2)	>100	>100	>100	>100	>100	>100	0.05 ^a^
*Staphylococcus aureus* (ATCC 6538)	>100	>100	>100	>100	6.25	6.25	1 ^b^
methicillin-resistant *S*. *aureus* (MRSAa)	>100	>100	100	>100	12.5	25	1 ^b^
*Bacillus subtilis* (ATCC 6633)	>100	>100	>100	>100	>100	>100	0.5 ^b^
*Pseudomonas aeruginosa* (PAO1)	>100	>100	>100	>100	>100	>100	1 ^c^
*Candida albicans* (SC 5314)	>100	>100	>100	>100	100	>100	0.016 ^d^

^a^ Isoniazid; ^b^ Vancomycin; ^c^ Ciprofloxacin; ^d^ Ketoconazole.

## Supplementary Materials

The following are available online at https://www.mdpi.com/1660-3397/17/5/262/s1, Figures S1–S8: 1D, 2D NMR, HRESIMS, UV and CD spectra of (±)-7,8-epoxy-brevianamide Q ((±)-**1**), Figures S9–S16: 1D, 2D NMR, HRESIMS, UV and CD spectra of (±)-8-hydroxy-brevianamide R ((±)-**2**), Figures S17–S23: 1D, 2D NMR, HRESIMS, UV and CD spectra of (±)-8-epihydroxy-brevianamide R ((±)-**3**).
