# Supplementary material for: New Diketopiperazines from a Marine-Derived Fungus Strain Aspergillus versicolor MF180151
Source: Mar Drugs. 2019 May 2;17(5):262. doi: 10.3390/md17050262 (PMC6562876; doi:10.3390/md17050262)
Supplement: Supplementary file 1 [file marinedrugs-17-00262-s001.pdf]

Supplementary Information

New diketopiperazines from a marine-derived fungus strain *Aspergillus versicolor* MF180151

Jiansen Hu[1,2], Zheng Li[1,2], Jieyu Gao[1,3], Hongtao He[1,2], Huanqin Dai[1], Xuekui Xia[4], Cuihua Liu[1*], Lixin Zhang[5*] and Fuhang Song[1*]

[1] CAS Key Laboratory of Pathogenic Microbiology and Immunology, Institute of Microbiology, Chinese Academy of Sciences, Beijing, 100101, China; huzxcv10@126.com (J.H.); bluewave2015@sina.com (Z.L.); hehongtao2010@live.cn (H.H.); huanqindai@gmail.com (H.D.);

[2] University of Chinese Academy of Sciences, Beijing, 100049, China;

[3] School of Food and Biological Engineering, Hefei University of Technology, Hefei, 230009, China; jieyu_gao@163.com (J.G.);

[4] Key Biosensor Laboratory of Shandong Provinde, Biology Institute, Qilu University of Technology (Shandong Academy of Sciences), Jinan, 250013, China; xiaxk@sdas.org (X.X.);

[5] State Key Laboratory of Bioreactor Engineering, East China University of Science of Technology, Shanghai, 200237, China;

***** Correspondence: liucuihua@im.ac.cn (C.L.); lxzhang@ecust.edu.cn (L.Z.); songfuhang@im.ac.cn (F.S.)
Tel.: +86 10 64806197 (C.L.); +86 21 64252575 (L.Z.); +86 10 64806058 (F.S.)

List of Supporting Information

Figure S1. ^{1}H NMR Spectrum of compounds (±)-**1** in DMSO-d_6 at 600 MHz.............3
Figure S2. ^{13}C NMR Spectrum of compounds (±)-**1** in DMSO-d_6 at 600 MHz............4
Figure S3. HSQC NMR Spectrum of compounds (±)-**1** in DMSO-d_6 5
Figure S4. HMBC NMR Spectrum of compounds (±)-**1** in DMSO-d_6 6
Figure S5. ^{1}H-^{1}H COSY NMR Spectrum of compounds (±)-**1** in DMSO-d_6...............7
Figure S6. ROESY NMR Spectrum of compounds (±)-**1** in DMSO-d_6........................8
Figure S7. HRESIMS and UV spectrum of compounds (±)-**1** in CH_3OH.....................9
Figure S8. Circular Dichroism spectrum of compounds (±)-**1** in CH_3OH 10
Figure S9. ^{1}H NMR Spectrum of compounds (±)-**2** in DMSO-d_6 at 600 MHz...........11
Figure S10. ^{13}C NMR Spectrum of compounds (±)-**2** in DMSO-d_6 at 600 MHz........12
Figure S11. HSQC NMR Spectrum of compounds (±)-**2** in DMSO-d_6 at 600 MHz ..13
Figure S12. HMBC NMR Spectrum of compounds (±)-**2** in DMSO-d_6 at 600 MHz .14
Figure S13. ^{1}H-^{1}H COSY NMR Spectrum of compounds (±)-**2** in DMSO-d_6............15
Figure S14. ROESY NMR Spectrum of compounds (±)-**2** in DMSO-d_6....................16
Figure S15. HRESIMS and UV spectrum of compounds (±)-**2** in CH_3OH.................17
Figure S16. Circular Dichroism spectrum of compounds (±)-**2** in CH_3OH 18
Figure S17. ^{1}H NMR Spectrum of compounds (±)-**3** in DMSO-d_6 at 600 MHz.........19
Figure S18. ^{13}C NMR Spectrum of compounds (±)-**3** in DMSO-d_6 at 600 MHz........20
Figure S19. HSQC NMR Spectrum of compounds (±)-**3** in DMSO-d_6 21
Figure S20. HMBC NMR Spectrum of compounds (±)-**3** in DMSO-d_6 22
Figure S21. ^{1}H-^{1}H COSY NMR Spectrum of compounds (±)-**3** in DMSO-d_6............23
Figure S22. ROESY NMR Spectrum of compounds (±)-**3** in DMSO-d_6....................24
Figure S23. HRESIMS and UV spectrum of compounds (±)-**3** in CH_3OH.................25
Figure S24. Circular Dichroism spectrum of compounds (±)-**3** in CH_3OH 26

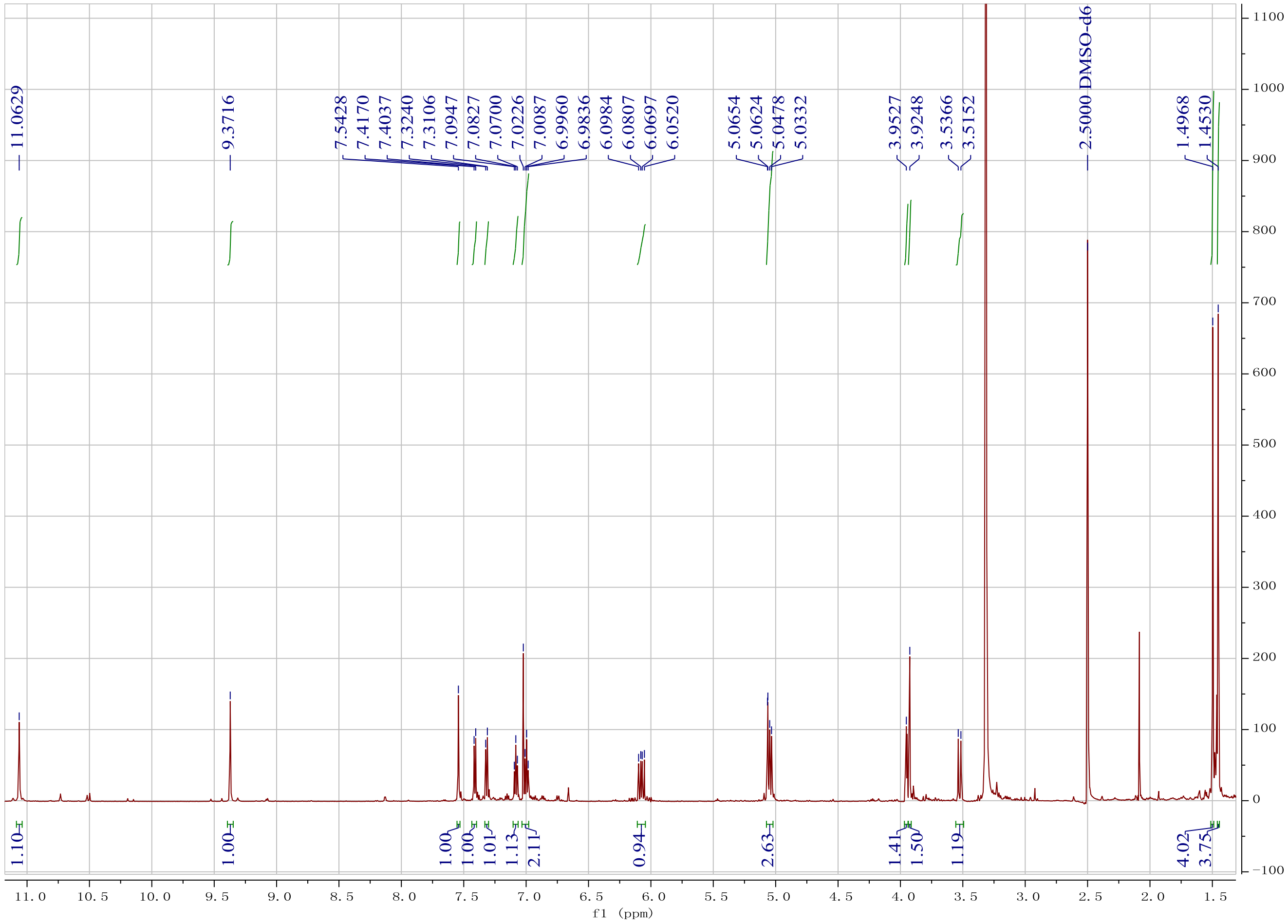

Figure S1. ^{1}H NMR Spectrum of compounds (±)-**1** in DMSO-d_6 at 600 MHz

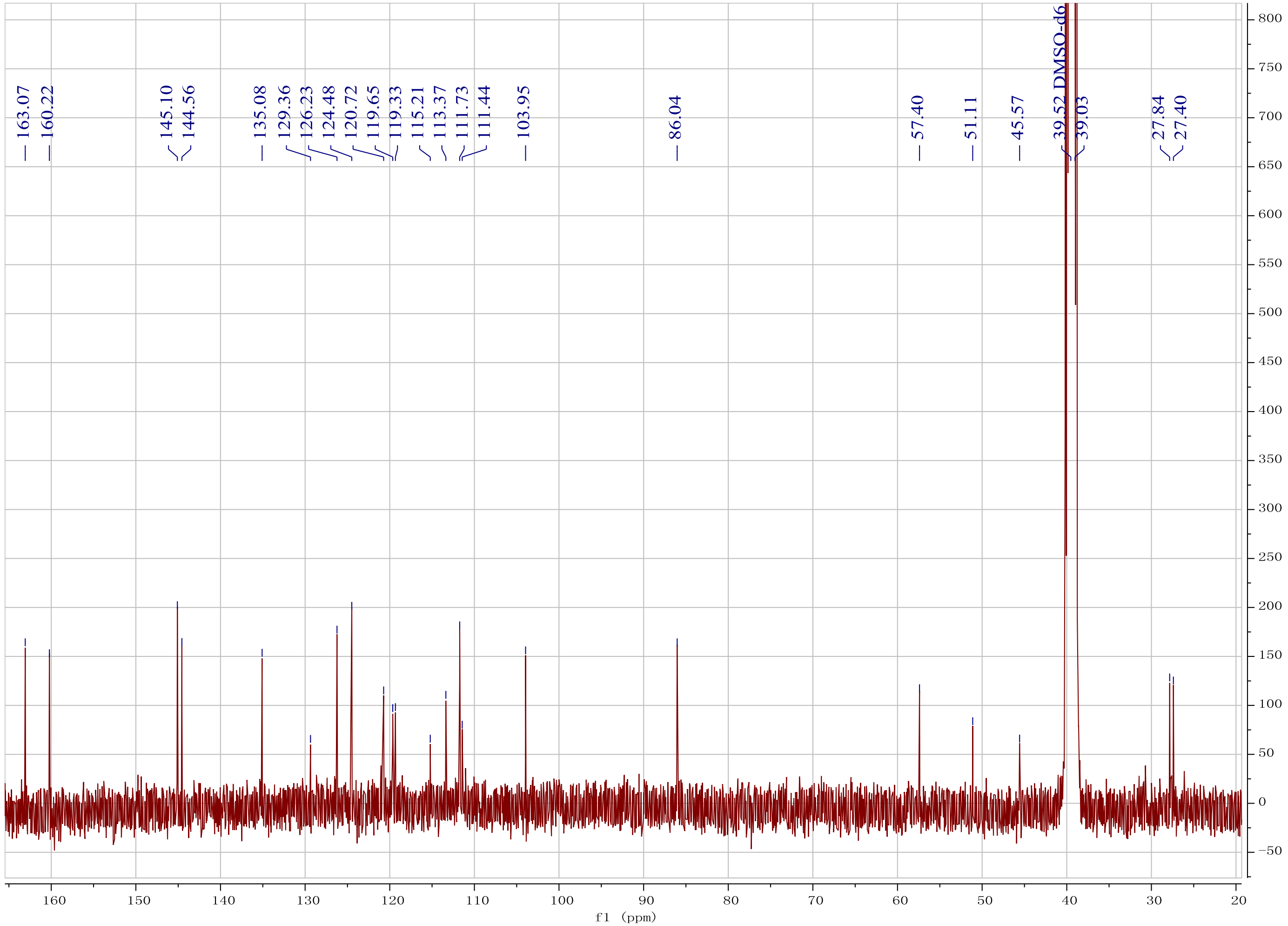

Figure S2. ^{13}C NMR Spectrum of compounds (±)-**1** in DMSO-d_6 at 600 MHz

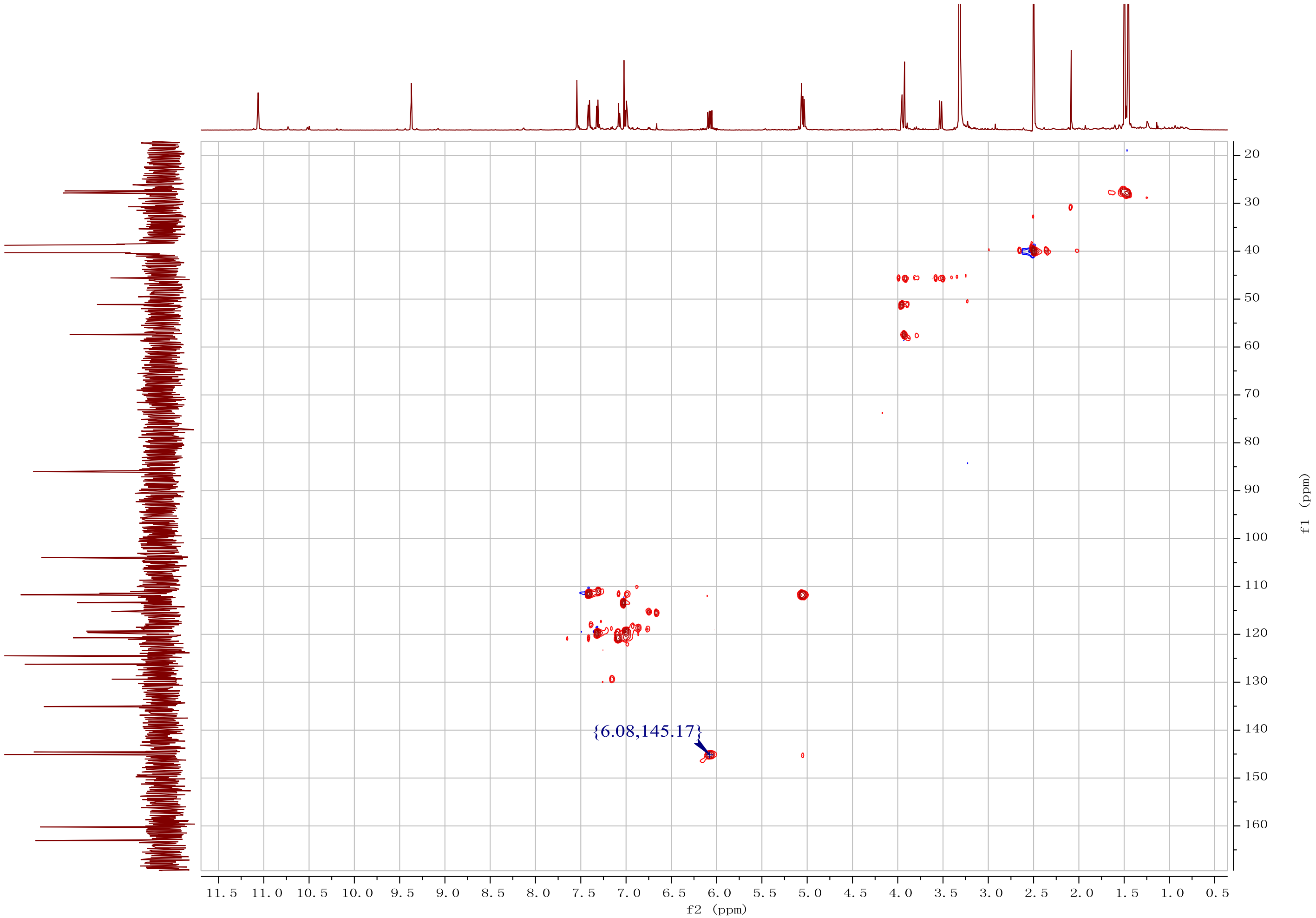

Figure S3. HSQC NMR Spectrum of compounds (±)-**1** in DMSO-d_6

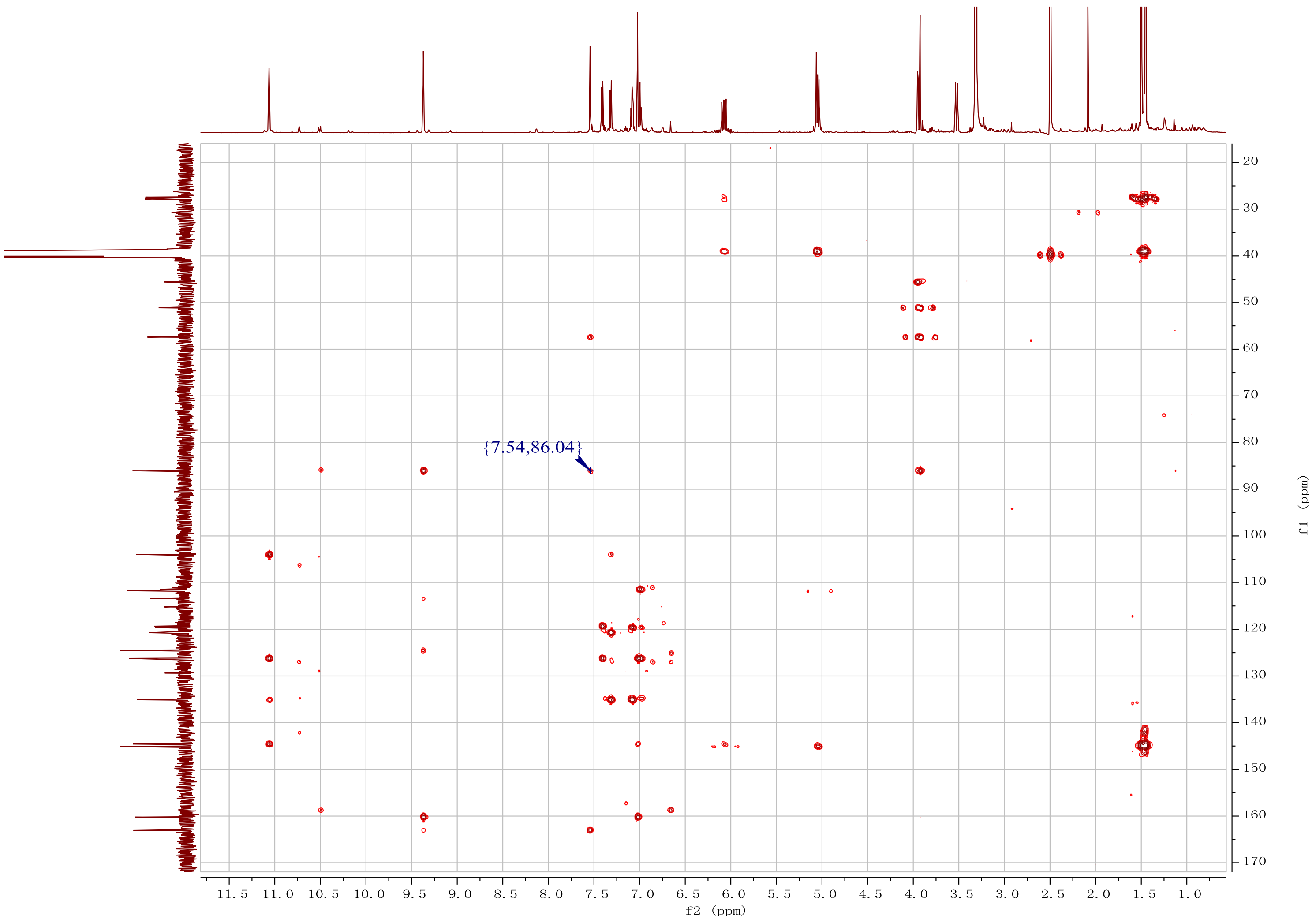

Figure S4. HMBC NMR Spectrum of compounds (±)-**1** in DMSO-d_6

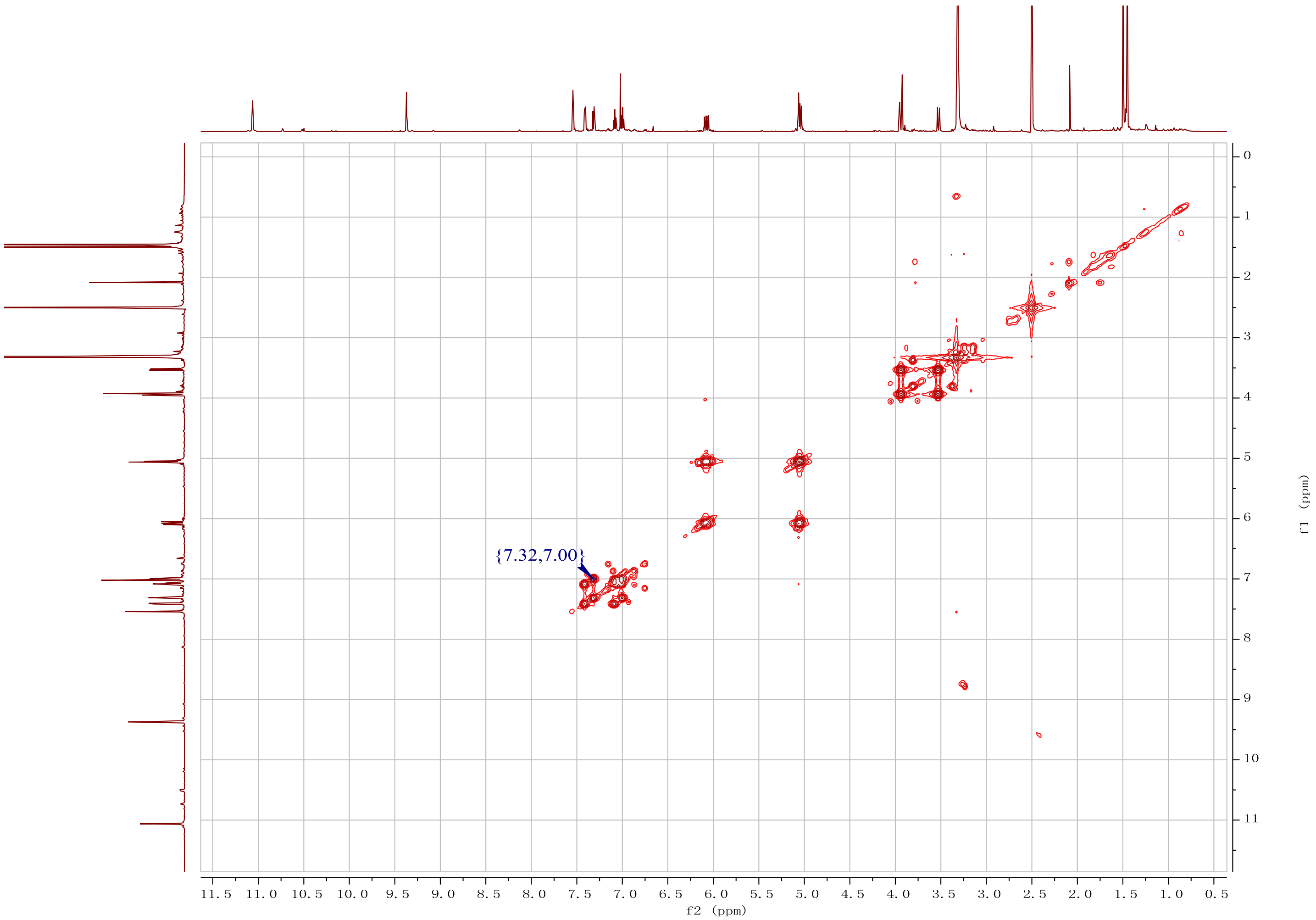

Figure S5. ^{1}H-^{1}H COSY NMR Spectrum of compounds (±)-**1** in DMSO-d_6

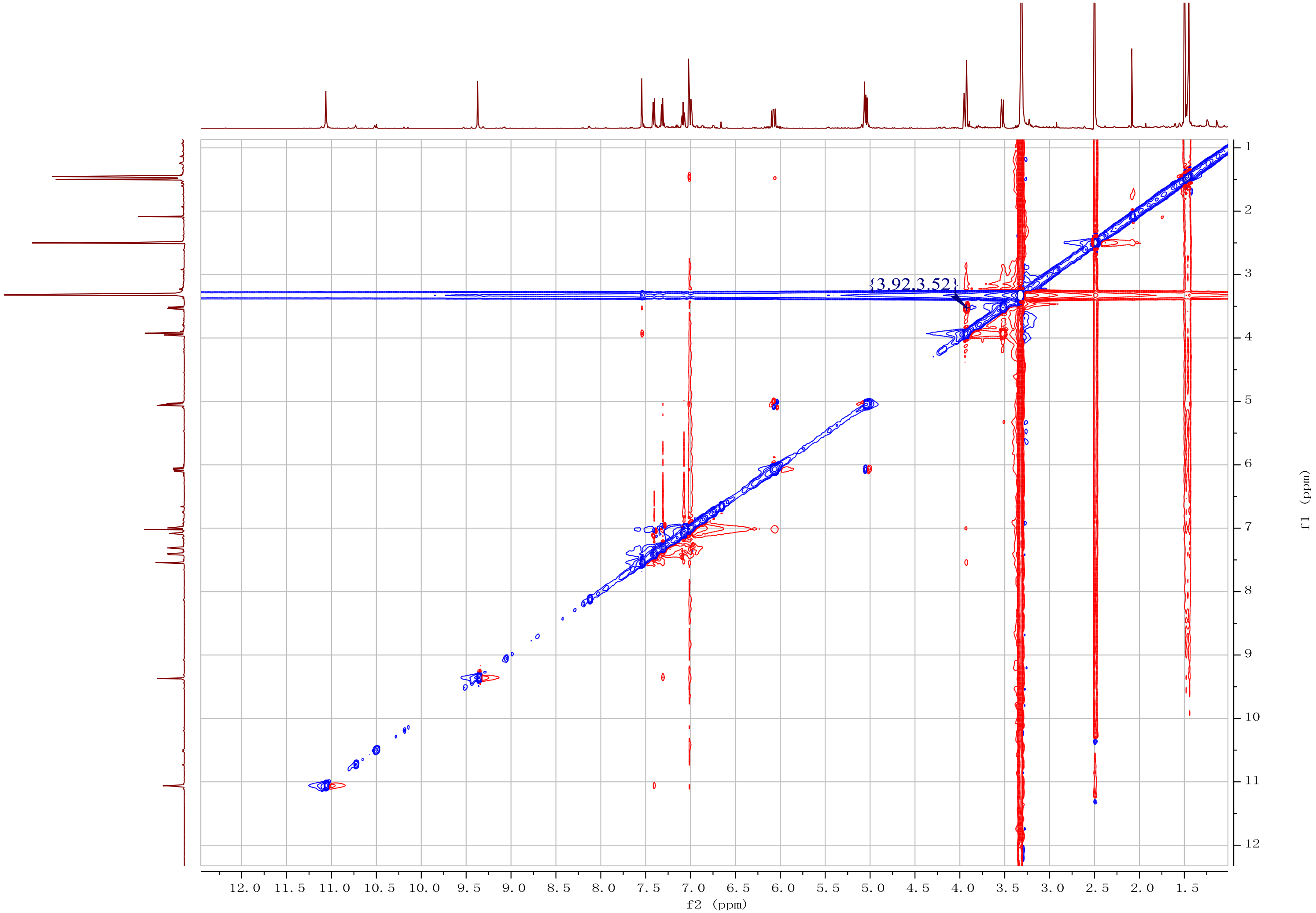

Figure S6. ROESY NMR Spectrum of compounds (±)-**1** in DMSO-d_6

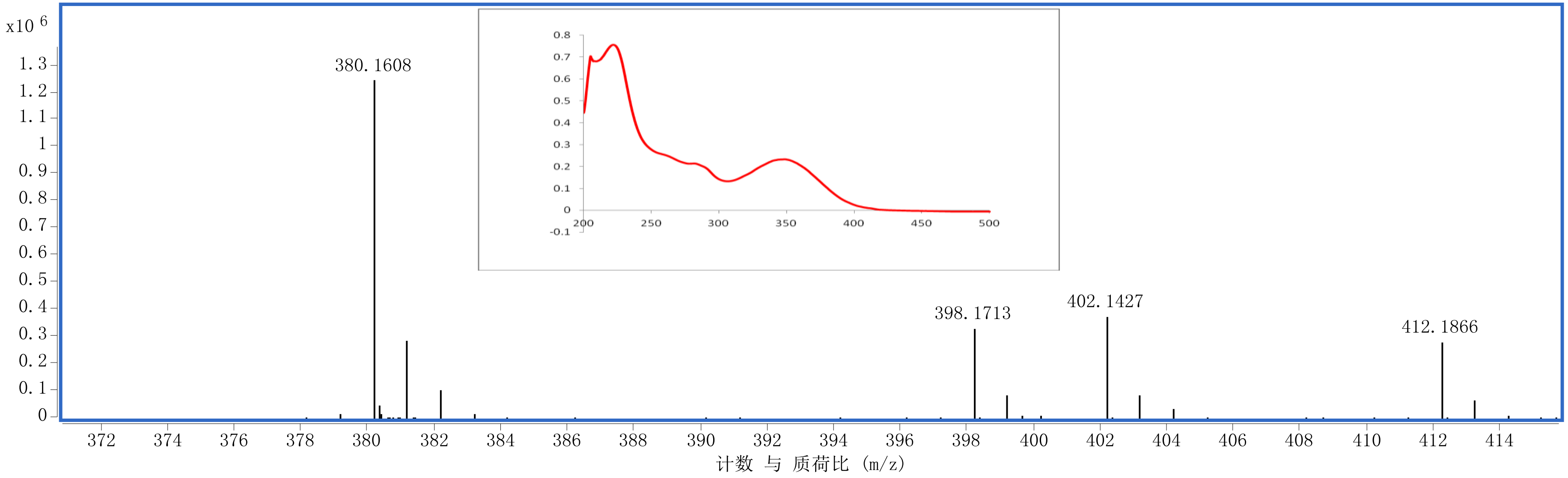

Figure S7. HRESIMS and UV spectrum of compounds (±)-**1** in CH_3OH

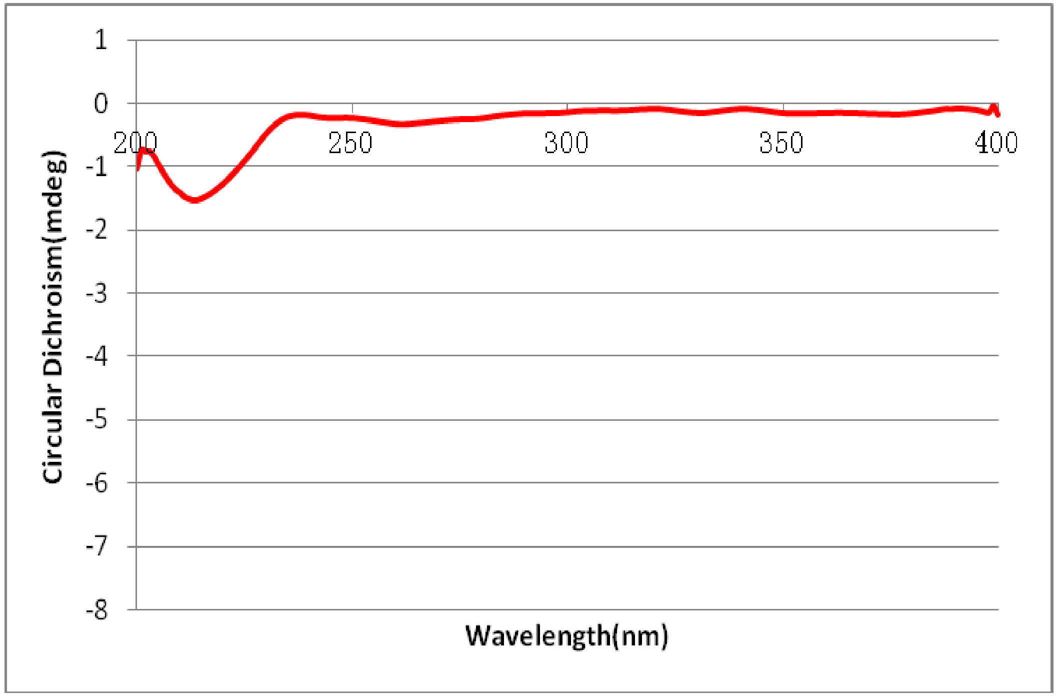

Figure S8. Circular Dichroism spectrum of compounds (±)-**1** in CH_3OH

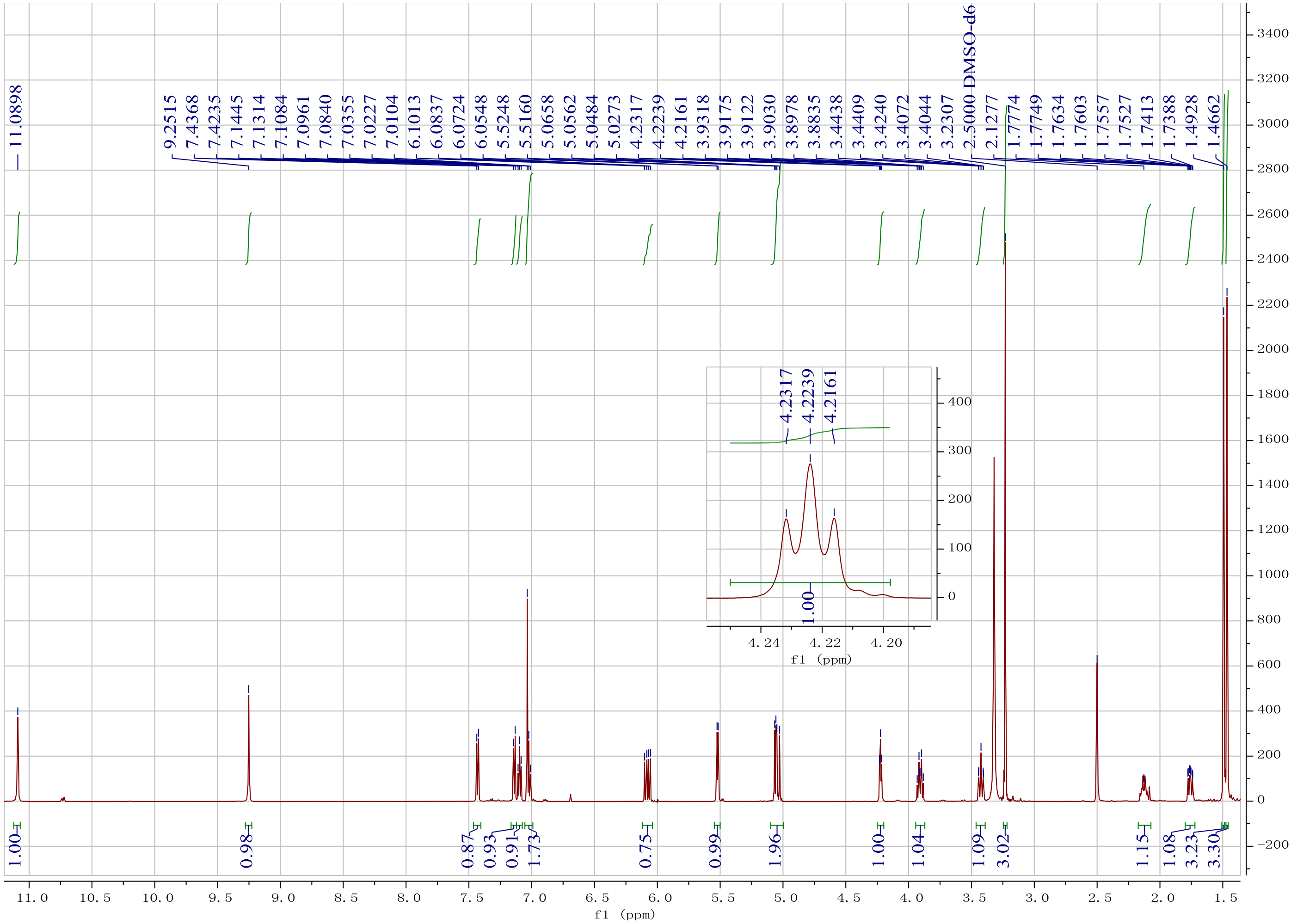

Figure S9. ^{1}H NMR Spectrum of compounds (±)-**2** in DMSO-d_6 at 600 MHz

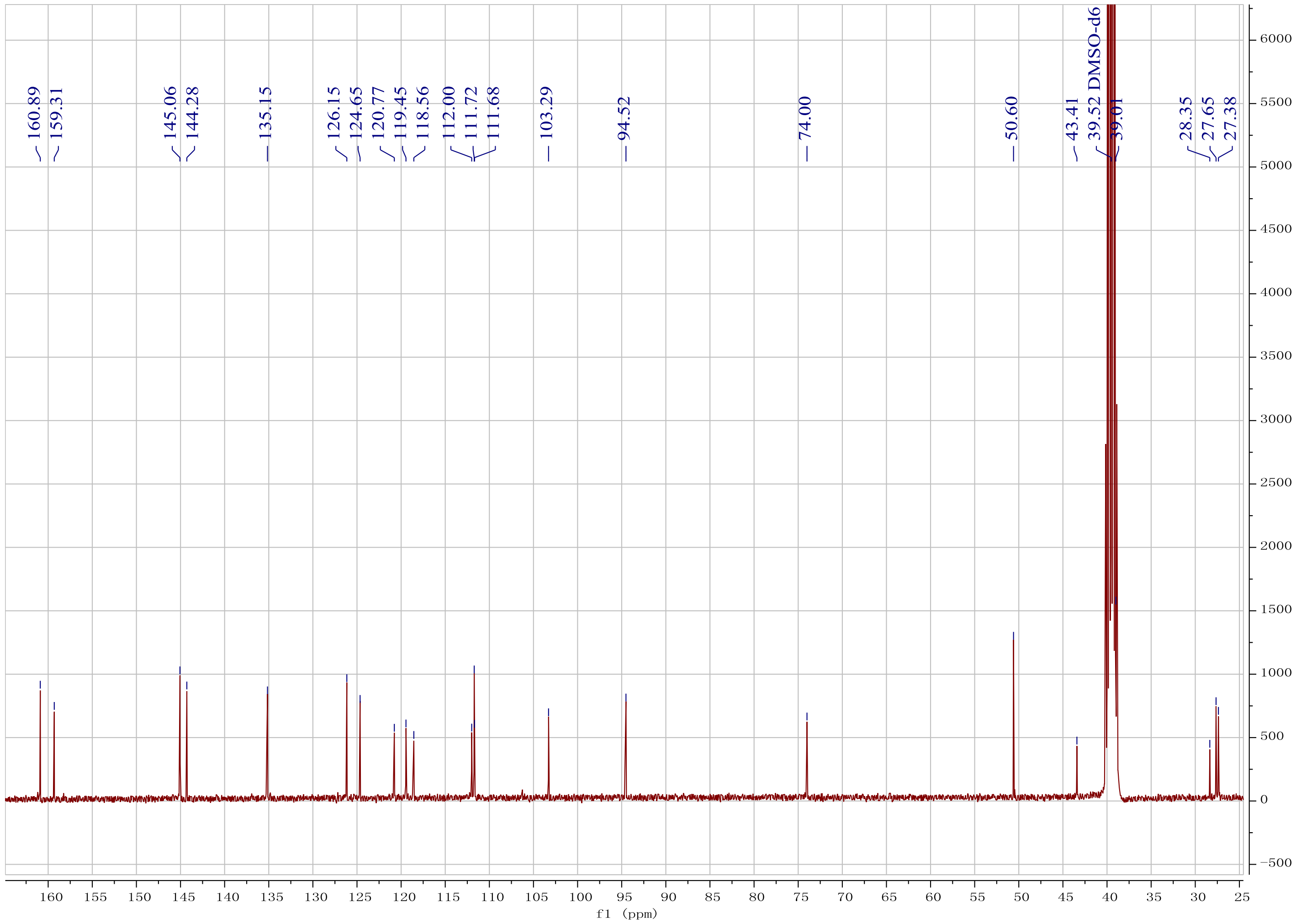

Figure S10. ^{13}C NMR Spectrum of compounds (±)-**2** in DMSO-d_6 at 600 MHz

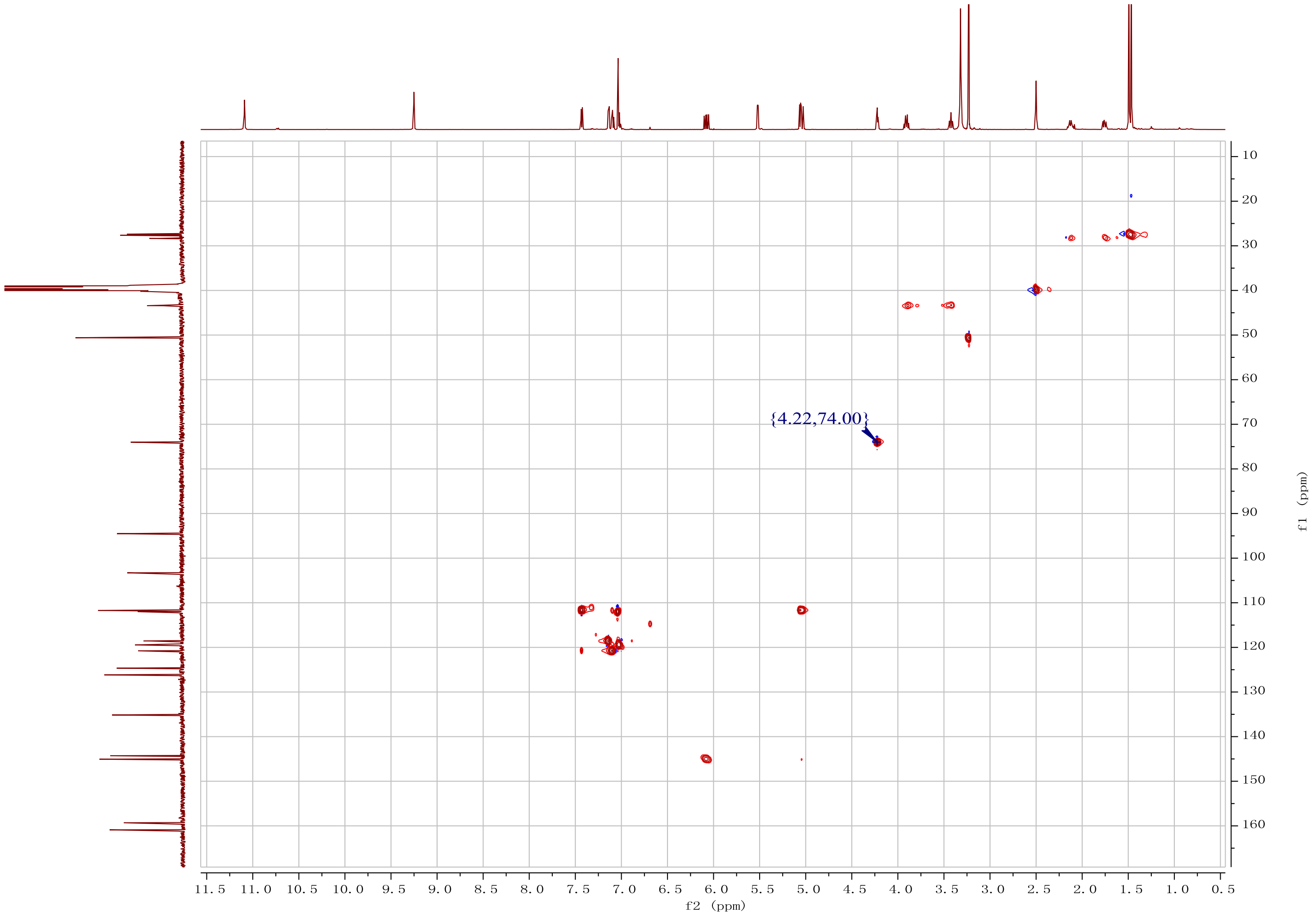

Figure S11. HSQC NMR Spectrum of compounds (±)-**2** in DMSO-d_6 at 600 MHz

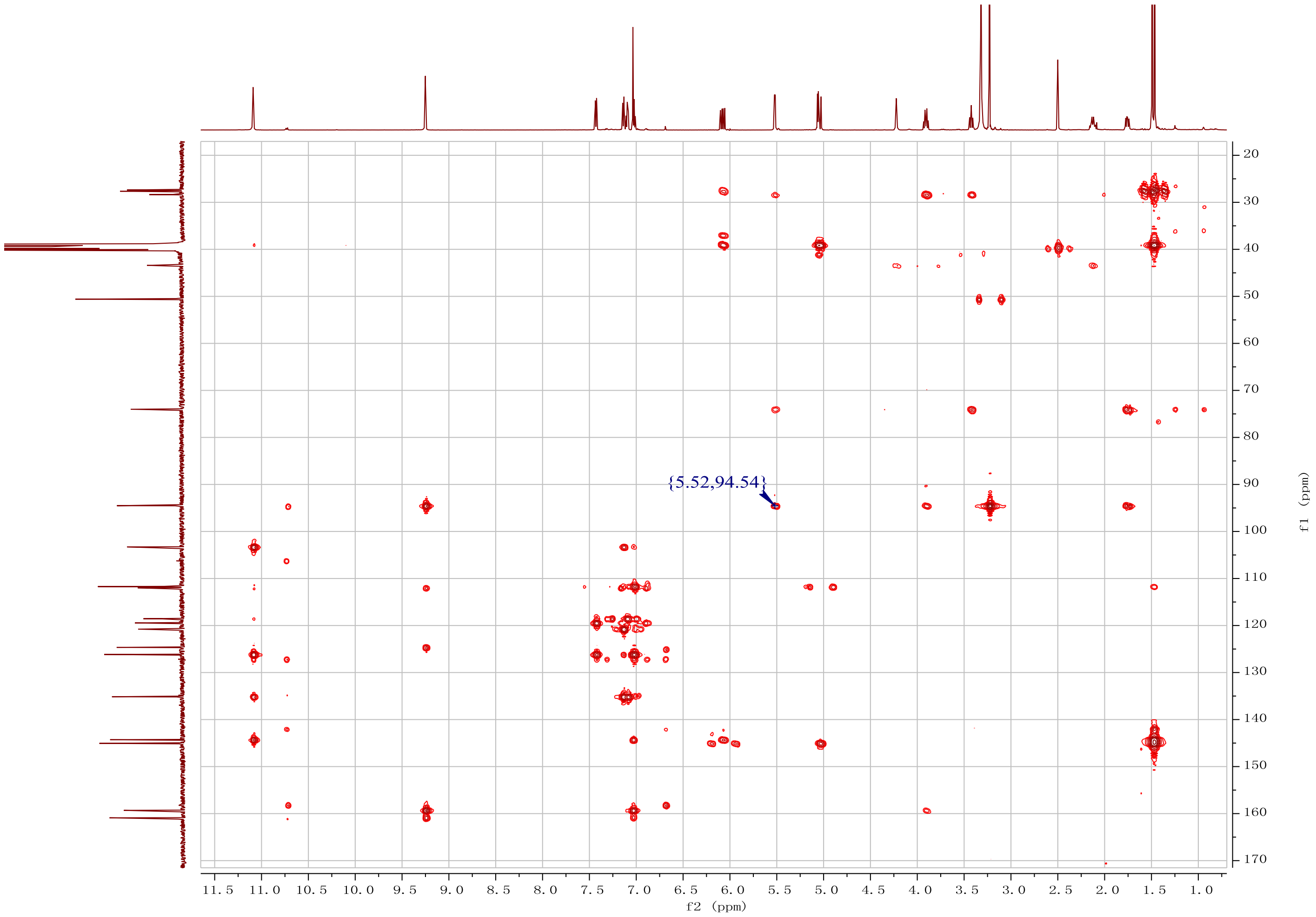

Figure S12. HMBC NMR Spectrum of compounds (±)-**2** in DMSO-d_6 at 600 MHz

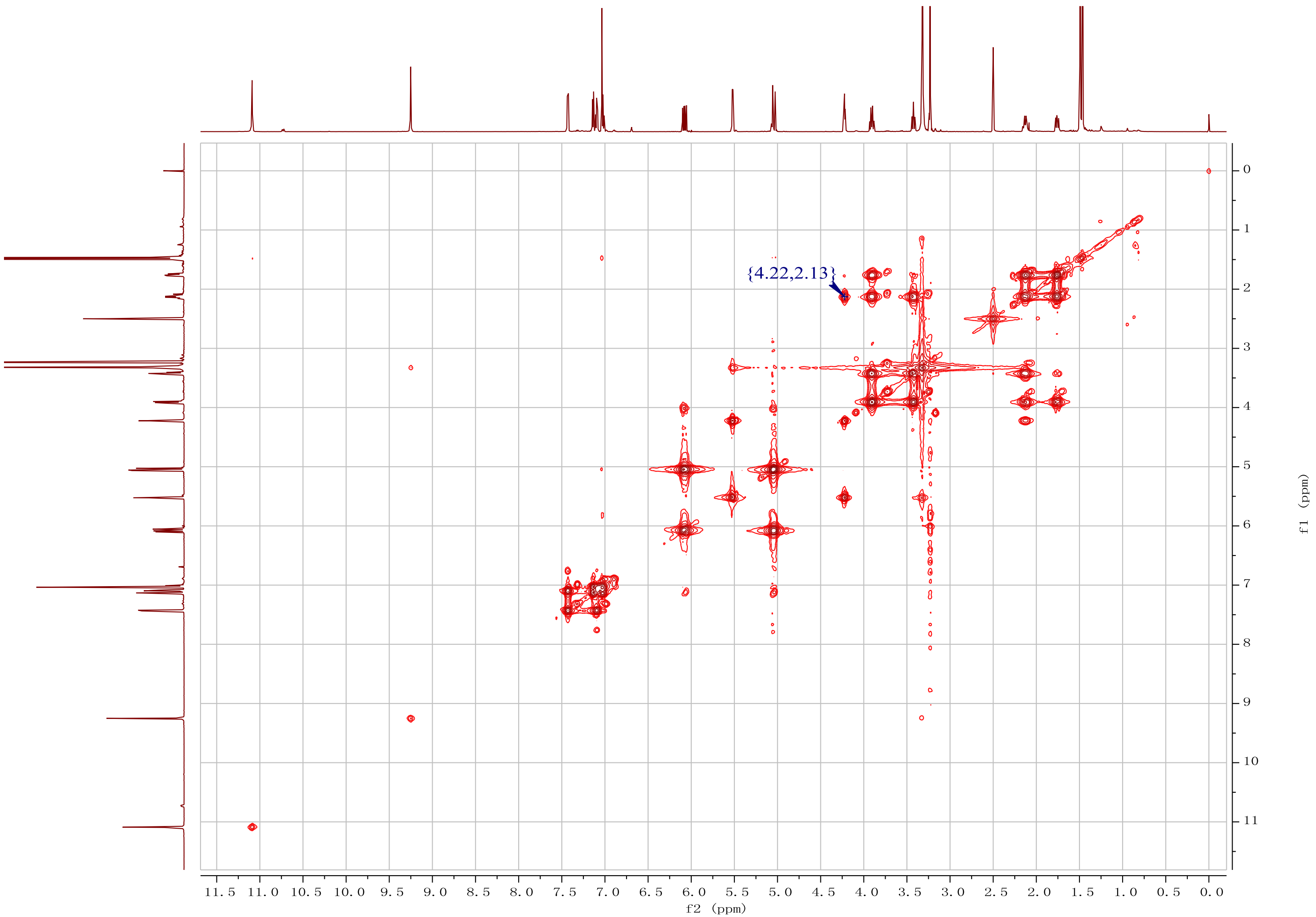

Figure S13. ^{1}H-^{1}H COSY NMR Spectrum of compounds (±)-**2** in DMSO-d_6

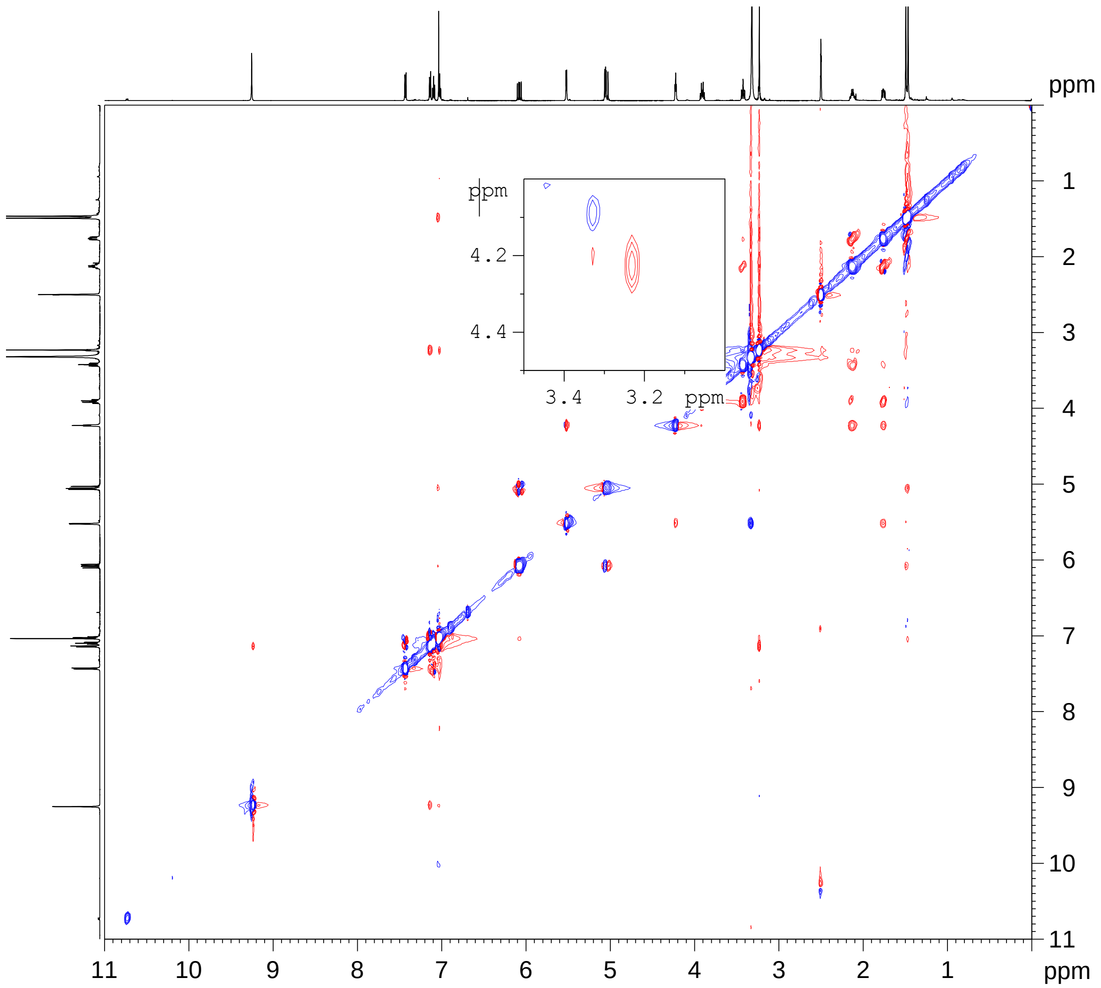

Figure S14. ROESY NMR Spectrum of compounds (±)-**2** in DMSO-d_6

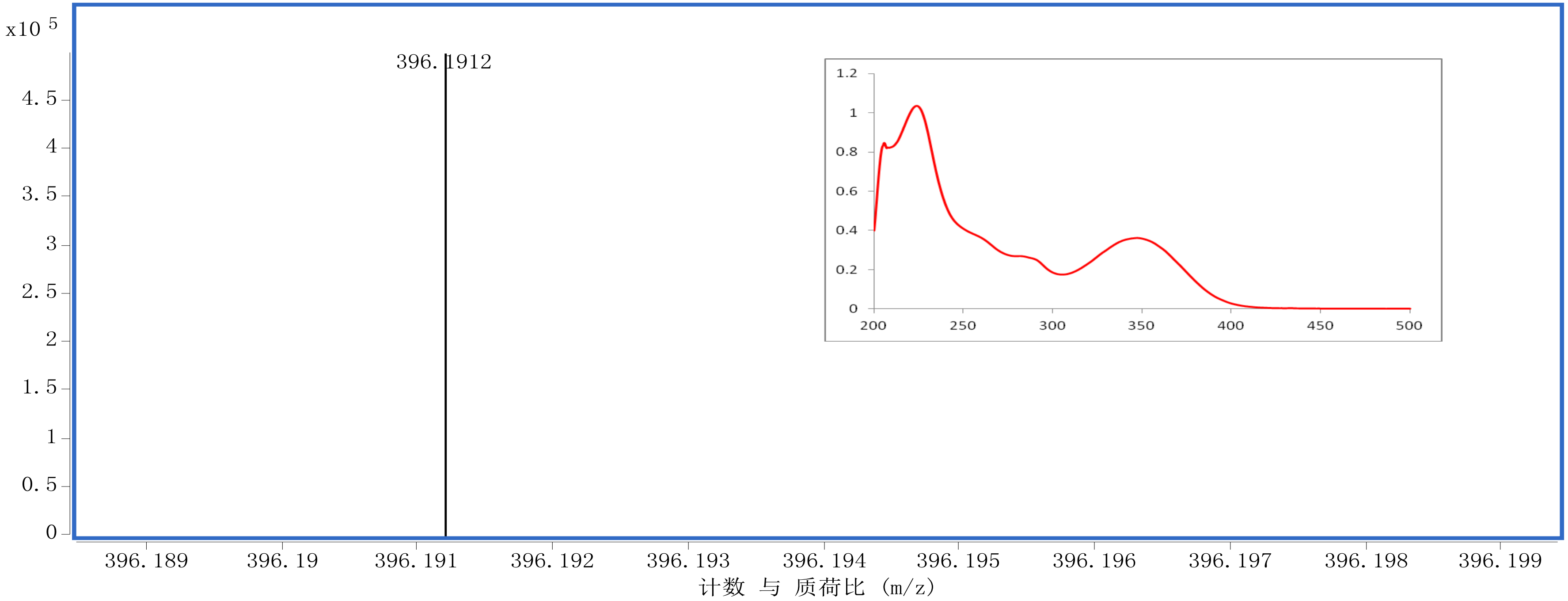

Figure S15. HRESIMS and UV spectrum of compounds (±)-**2** in CH_3OH

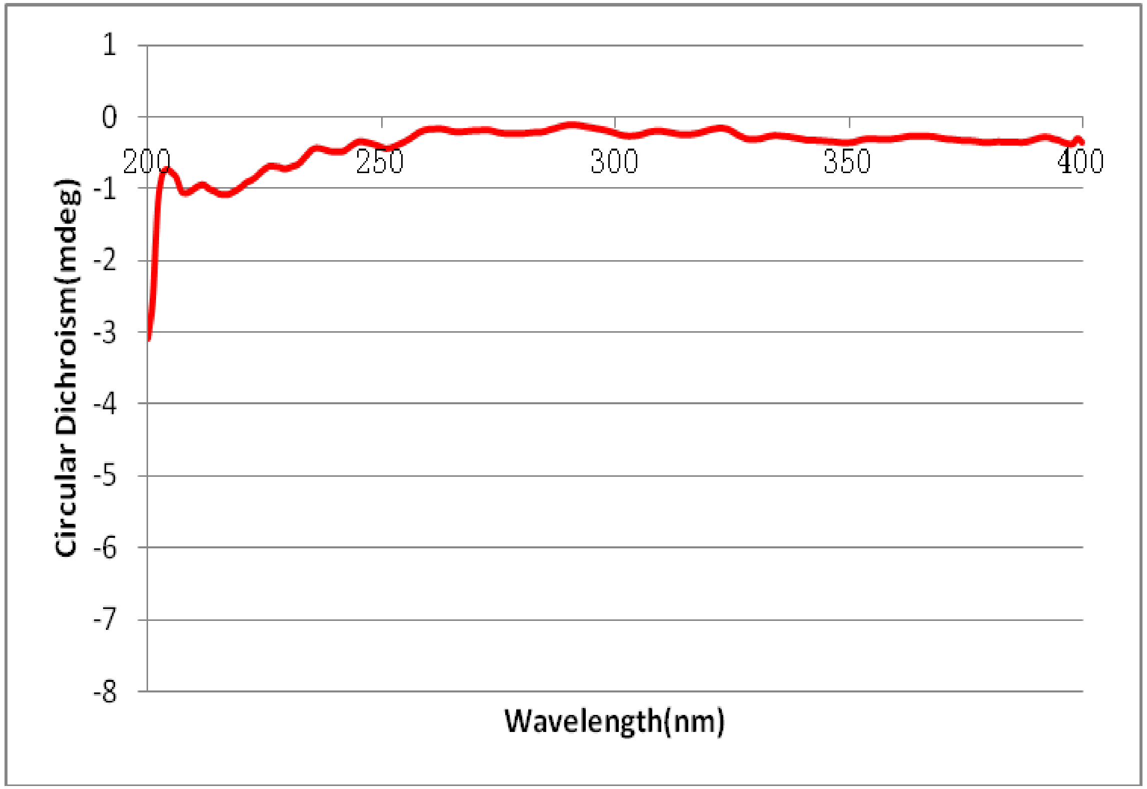

Figure S16. Circular Dichroism spectrum of compounds (±)-**2** in CH_3OH

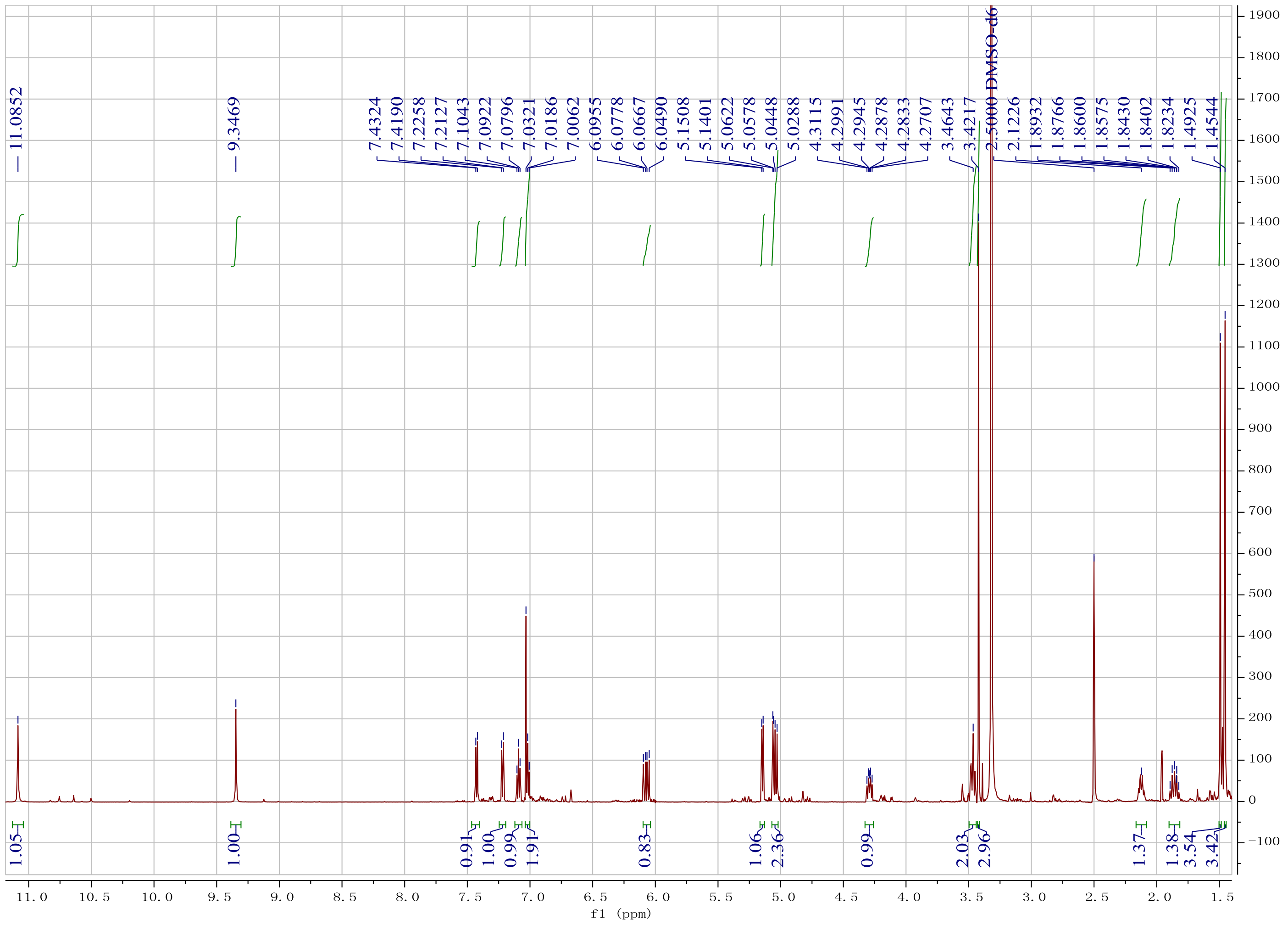

Figure S17. ^{1}H NMR Spectrum of compounds (±)-**3** in DMSO-d_6 at 600 MHz

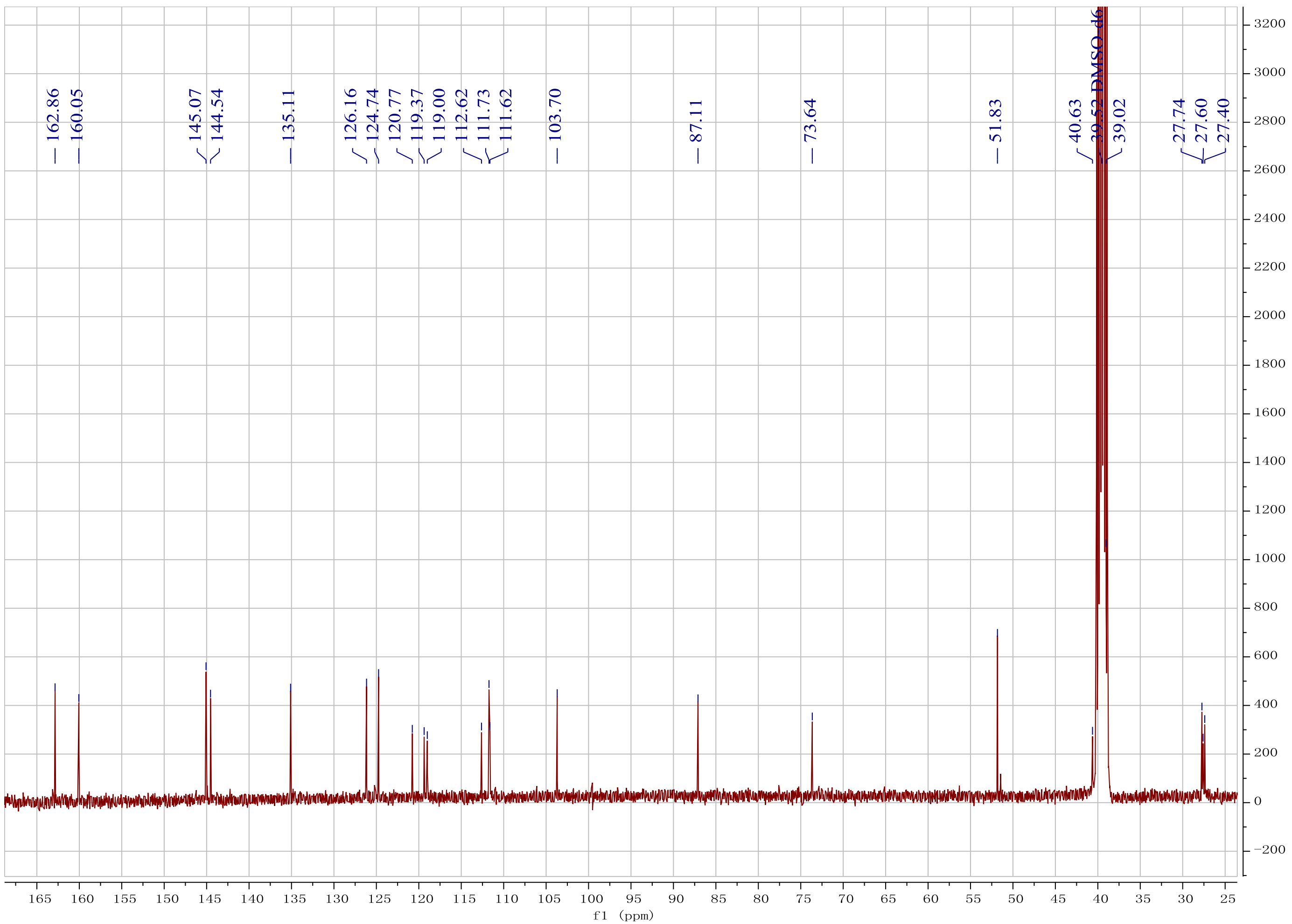

Figure S18. ^{13}C NMR Spectrum of compounds (±)-**3** in DMSO-d_6 at 600 MHz

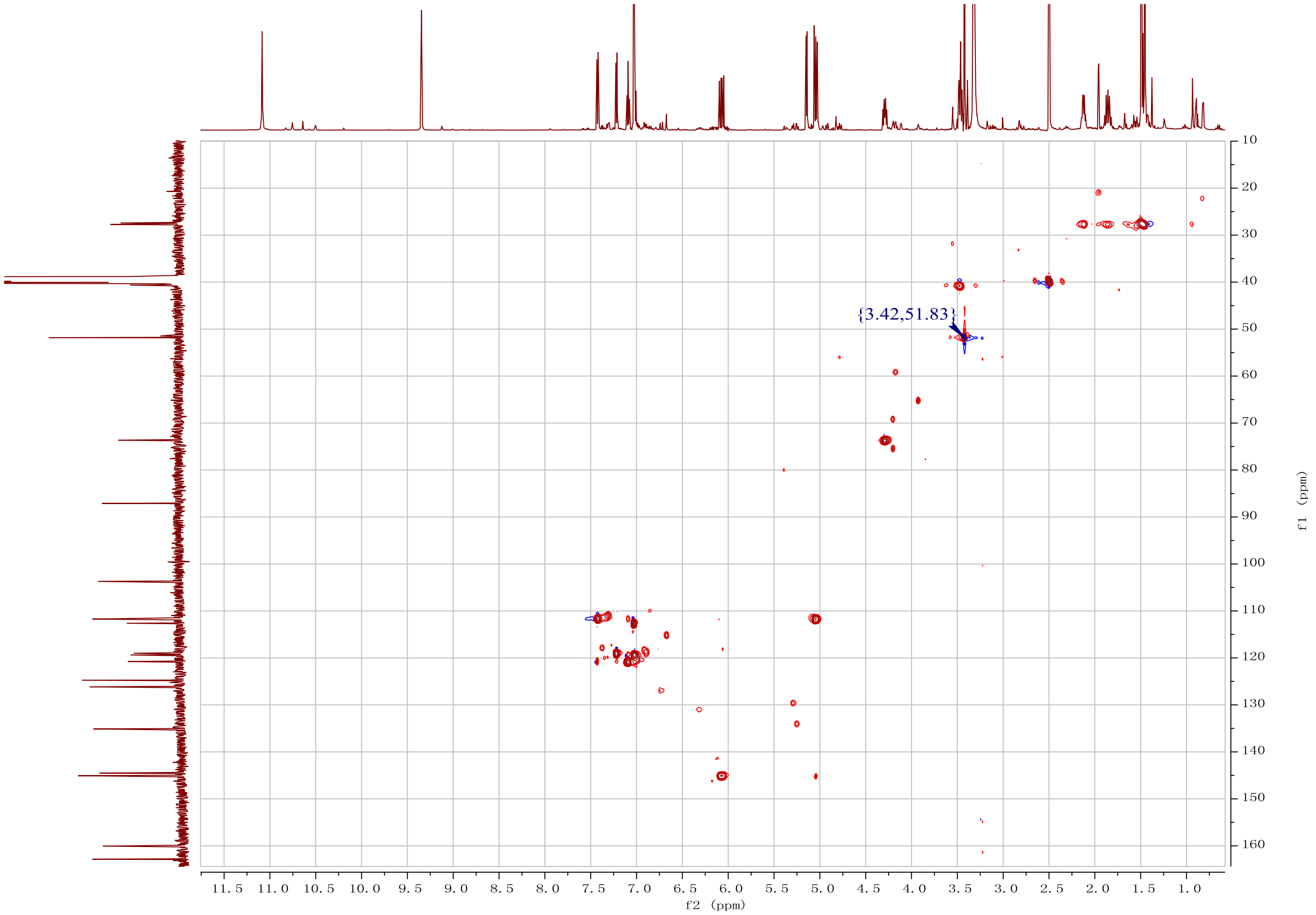

Figure S19. HSQC NMR Spectrum of compounds (±)-**3** in DMSO-d_6

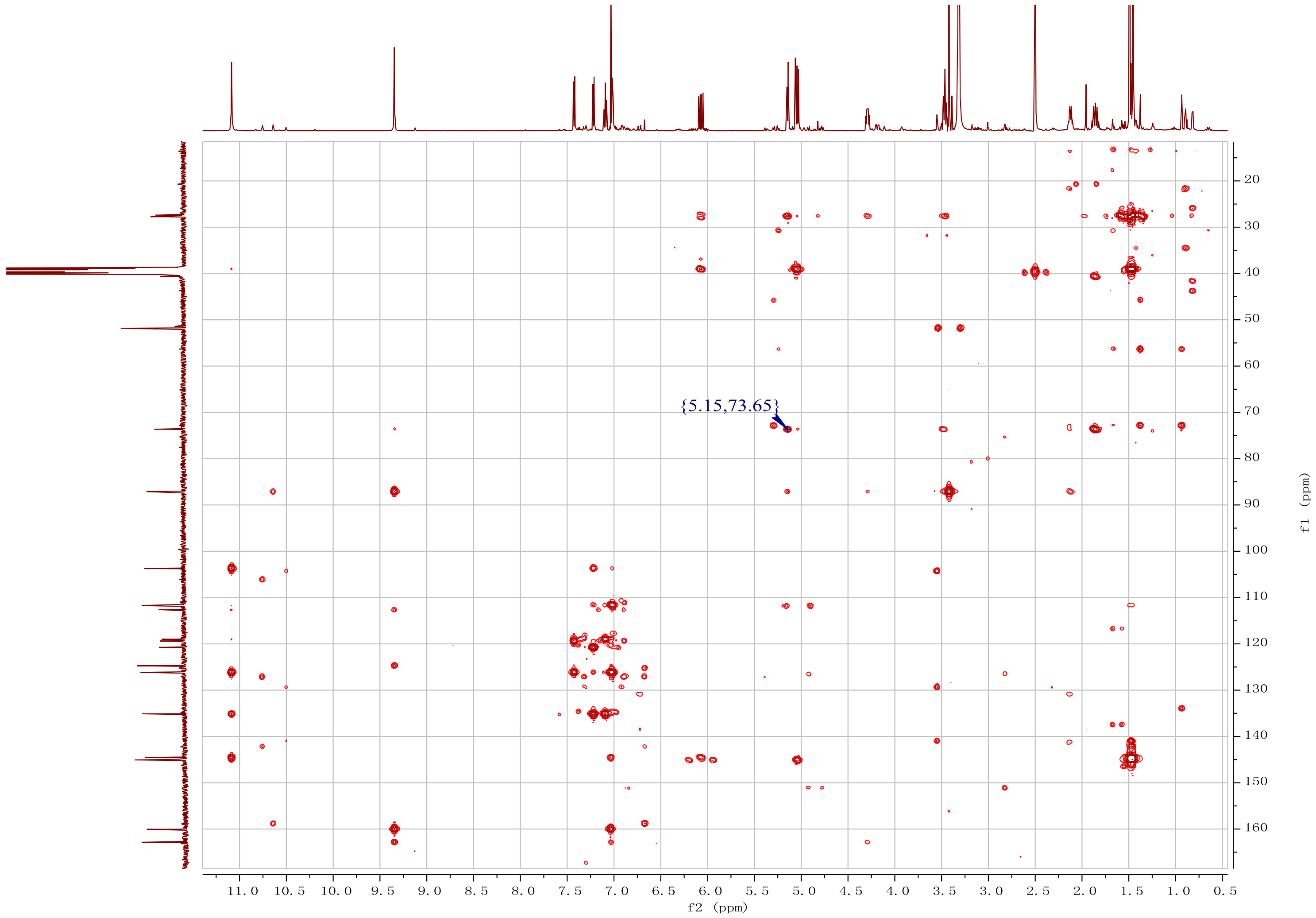

Figure S20. HMBC NMR Spectrum of compounds (±)-**3** in DMSO-d_6

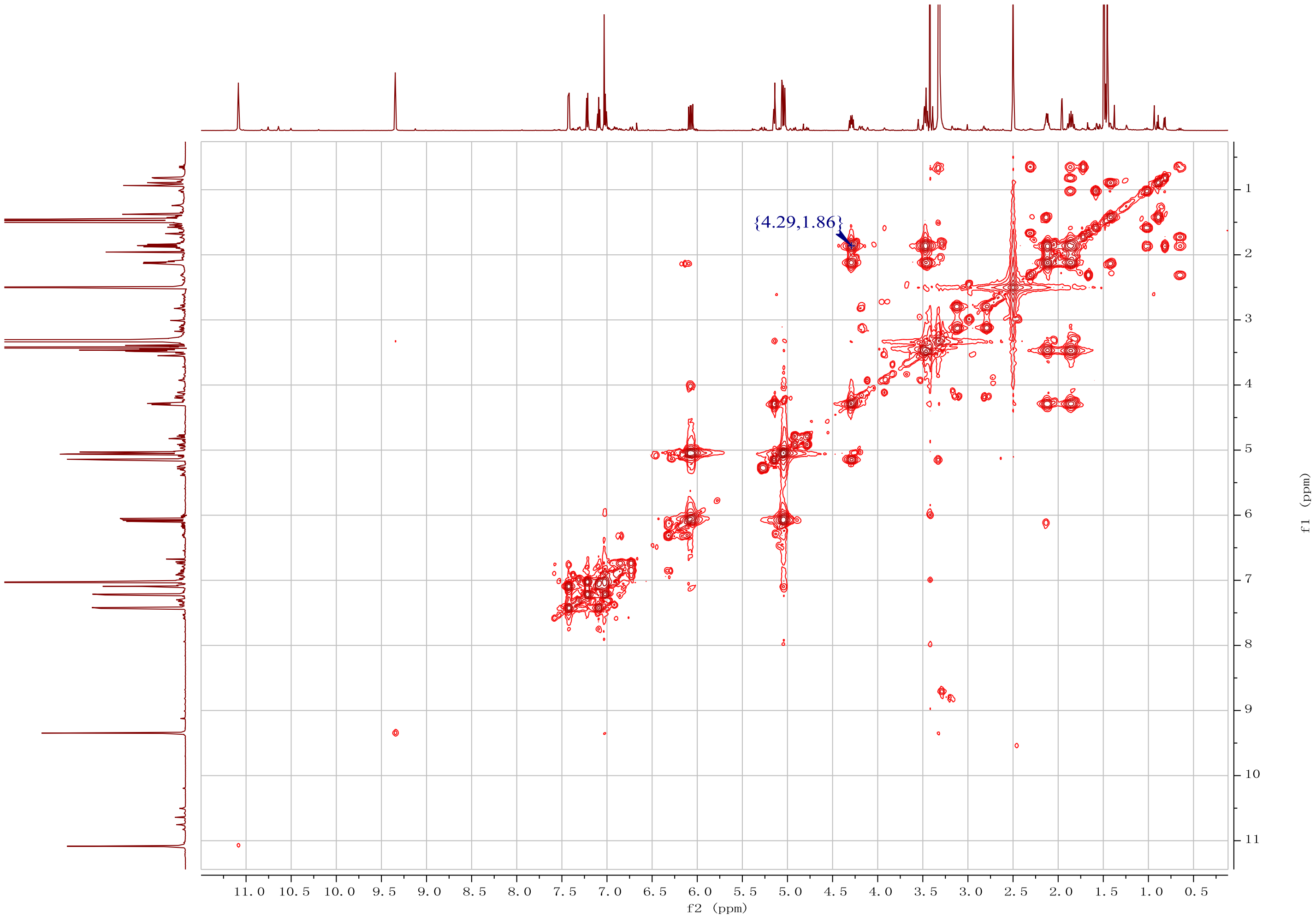

Figure S21. ^{1}H-^{1}H COSY NMR Spectrum of compounds (±)-**3** in DMSO-d_6

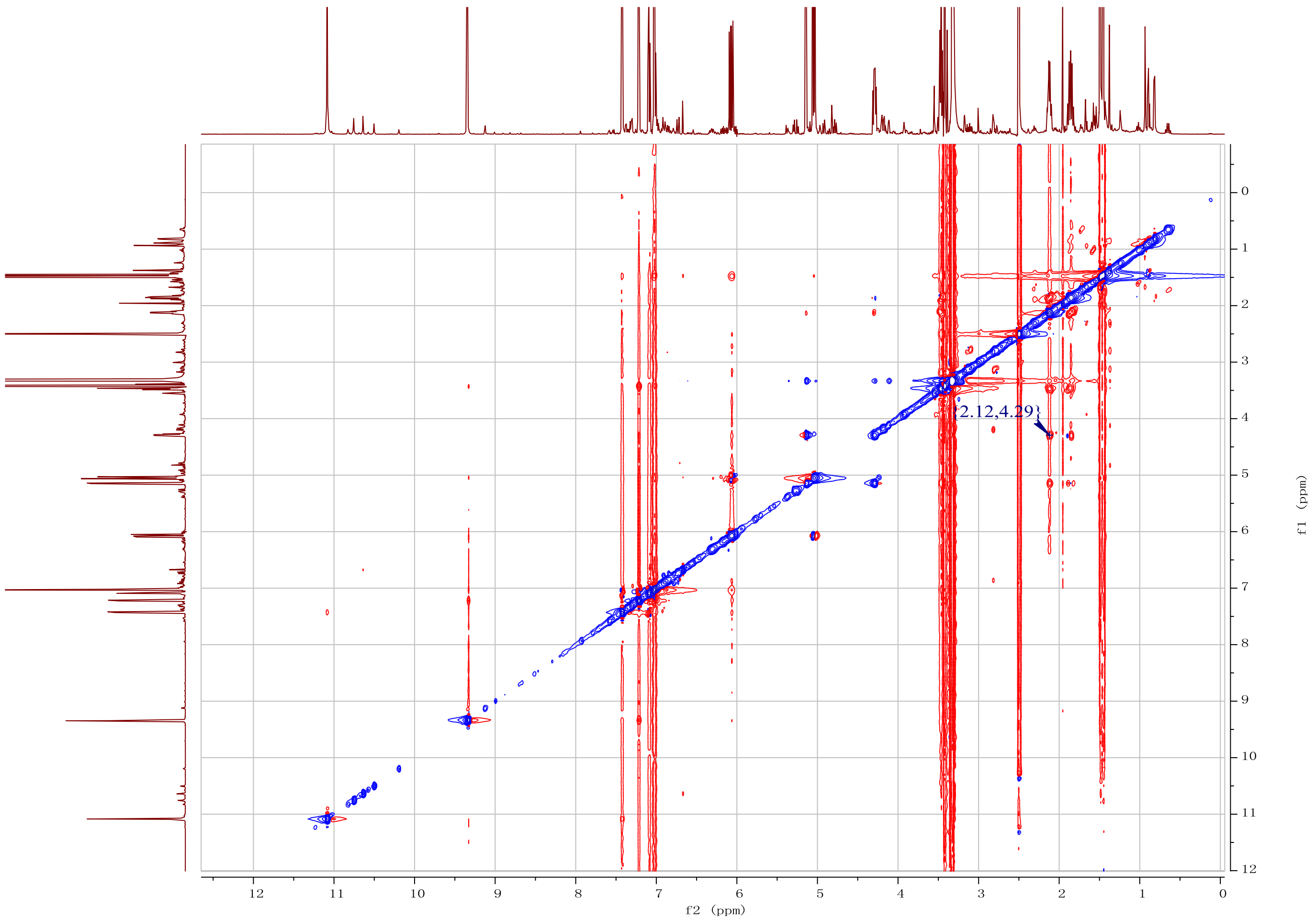

Figure S22. ROESY NMR Spectrum of compounds (±)-**3** in DMSO-d_6

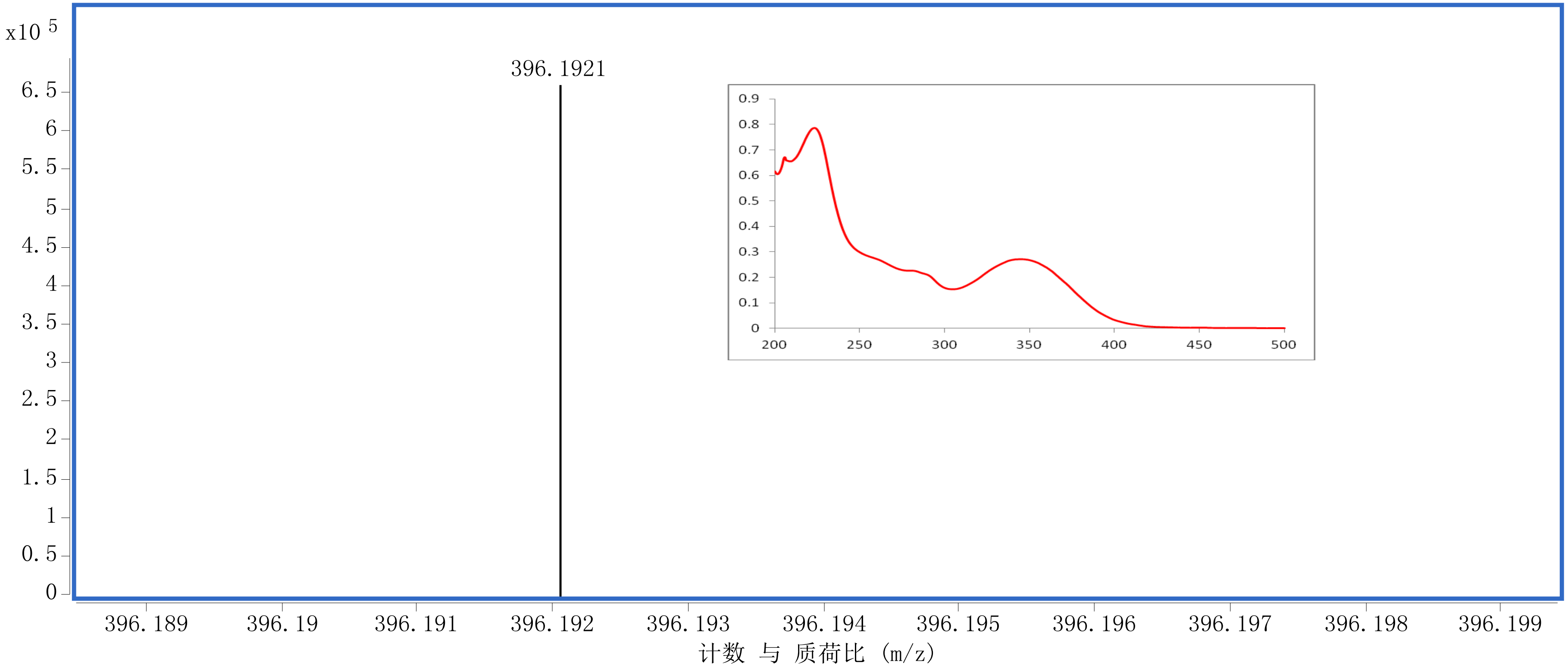

Figure S23. HRESIMS and UV spectrum of compounds (±)-**3** in CH_3OH

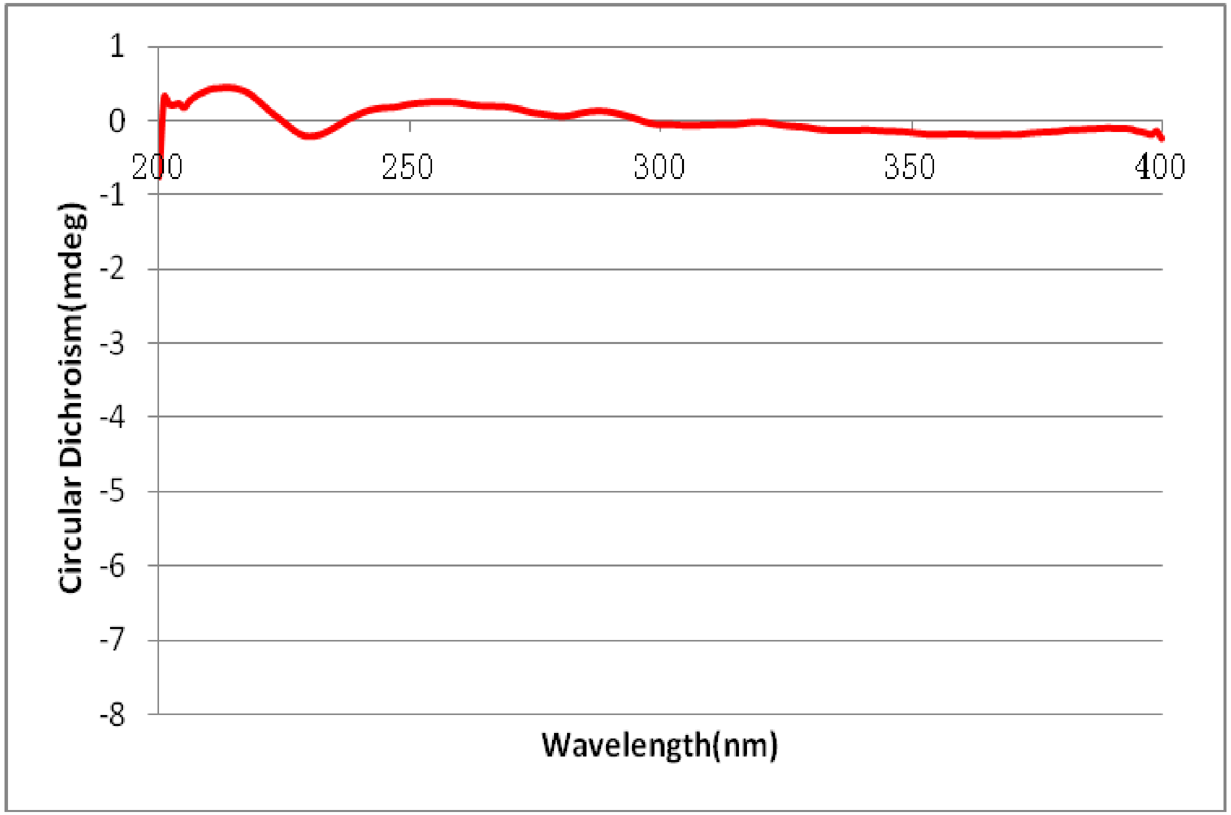

Figure S24. Circular Dichroism spectrum of compounds (±)-**3** in CH_3OH
